# A randomised controlled trial of the 5:2 diet

**DOI:** 10.1371/journal.pone.0258853

**Published:** 2021-11-17

**Authors:** Peter Hajek, Dunja Przulj, Francesca Pesola, Hayden McRobbie, Sarrah Peerbux, Anna Phillips-Waller, Natalie Bisal, Katie Myers Smith

**Affiliations:** Health and Lifestyle Research Unit, Wolfson Institute of Population Health, Queen Mary University of London, London, United Kingdom; Weill Cornell Medical College in Qatar, QATAR

## Abstract

**Objective:**

The 5:2 diet is a popular intermittent energy restriction method of weight management that awaits further evaluation. We compared the effects of one-off 5:2 instructions with the effects of one-off standard multicomponent weight-management advice; and also examined whether additional behavioural support enhances 5:2 adherence and efficacy compared to one-off instructions.

**Methods:**

Three hundred adults with obesity were randomised to receive a Standard Brief Advice (SBA) covering diet and physical activity (N = 100); 5:2 self-help instructions (5:2SH) (N = 100); or 5:2SH plus six once-weekly group support sessions (N = 100). Participants were followed up for one year.

**Results:**

Adherence to 5:2SH was initially high (74% at 6 weeks), but it declined over time (31% at 6 months and 22% at one year). 5:2SH and SBA achieved similar weight-loss at six months (-1.8kg (SD = 3.5) vs -1.7kg (SD = 4.4); b = 0.23, 95%CI:-0.79–1.27, p = 0.7) and at one year (-1.9kg (SD = 4.9) vs -1.8kg (SD = 5.7), b = 0.20, 95%CI:-1.21–1.60, p = 0.79), with 18% vs 15% participants losing ≥5% of their body weight with 5:2SH and SBA, respectively at one year (RR = 0.83, 95%CI:0.44–1.54, p = 0.55). Both interventions received positive ratings, but 5:2SH ratings were significantly higher. 5:2SH had no negative effect on fat and fiber intake and physical activity compared to SBA. Compared to 5:2SH, 5:2G generated a greater weight loss at 6 weeks (-2.3kg vs -1.5kg; b = 0.74, 95%CI:1.37–0.11, p = 0.02), but by one year, the difference was no longer significant (-2.6kg vs -1.9kg, p = 0.37; ≥5% body weight loss 28% vs 18%, p = 0.10).

**Conclusions:**

Simple 5:2 advice and multicomponent weight management advice generated similar modest results. The 5:2 diet did not undermine other health behaviours, and it received more favourable ratings. Adding initial group support enhanced 5:2 adherence and effects, but the impact diminished over time. Health professionals who provide brief weight management advice may consider including the 5:2 advice as an option.

**Trial registration:**

ISRCTN registry (ISRCTN79408248).

## Introduction

The prevalence of obesity is high worldwide [1], especially among disadvantaged socioeconomic groups in high-income countries [2, 3]. Obesity is associated with a number of adverse health conditions that long-term weight loss can improve [4].

Low energy formula diets can be effective in helping people lose weight [5]. E.g. a total diet replacement provided free of charge together with intensive support to persons with diabetes was shown remarkably effective (mean weight loss of 10 kg at one year, with 24% of participants losing 15 kg or more) [6]. Sustained dieting without such external support however is difficult for most people, as shown by a typical trajectory of weight regain [7]. There remains a need for a broader range of weight loss interventions that can be disseminated easily to the wider population.

A type of intermittent energy restriction (IER) weight loss intervention called the 5:2 diet (5:2) was made popular in the UK by a BBC Horizon documentary screened in August 2012 [8], followed by a bestselling book [9]. The approach was a development of previous work on intermittent fasting by e.g. Harvie and Howell [10]. Dieters restrict their caloric intake on two non-consecutive days a week (to 500 kcal for women and 600 kcal for men), with sensible eating but no formal energy restrictions on the remaining days [8, 10]. The diet has attracted much attention internationally and it remains highly popular.

IER is not a new concept. The first trial of IER was published 35 years ago [11]. Narrative reviews of IER [12–14] draw heavily on animal data and highlight the paucity of definitive human trials, but several studies evaluated stricter versions of IER in humans [11, 15–27]. Typically, participants were asked to restrict energy to 25% of their usual daily calories every other day (‘alternate day fasting’) or on two consecutive days per week [28] sometimes after a period on a very-low energy diet. Participants were usually also asked to use prescriptive diets on non-fasting days. IER had similar effects to continuous energy restriction (CER) on weight loss, body composition and metabolic markers and in some studies it had better effects on e.g. insulin sensitivity and lipid profiles [15–18]. One review found a better weight loss with IER in the short term [28] while other meta analyses and systematic reviews found IER achieving similar results as CER [29–31]. However, these traditional versions of IER that require relatively severe fasting did not have a wide appeal and did not translate into clinical practice.

The main advantages of the 5:2 diet that appear to be responsible for its popularity are its simplicity, the recommendation that fasting takes place on just two separate days (‘going hungry’ for two consecutive days or every other day seems too difficult for most people), and the removal of food restrictions on non-fasting days [9]. Unlike most other approaches to weight loss, 5–2 does not require relentless self-control and allows dieters to stop worrying about food intake on five days a week. Compared to the previous stricter approaches, this can be expected to have a weaker effect, but this downside could in theory be compensated for by better adherence and maintenance of the intervention.

The approach also holds an additional promise in that if it is effective, it could be particularly helpful for disadvantaged groups. Most existing weight management programmes include complex information on nutrition, caloric content of food, coping strategies, behavioural tasks, food diaries, exercise etc. They also usually incur costs, e.g. of commercial diet replacements, and require extensive lifestyle changes. All of these requirements are difficult to process and implement even for people with good socioeconomic resources and well organised lives and routines. Most studies in this field involve participants from middle to high socioeconomic groups, and typically report limited adherence and only modest weight loss [33]. The fact that 5:2 is much more straightforward and less demanding could make it particularly promising for people with high levels of stress, high incidence of unpredictable events, and limited resources. Even 5:2 poses significant demands on the fasting days though, and it is possible that overall adherence to it is as low as adherence to other approaches.

In terms of intervention reach, the ease of delivery of the 5:2 instructions means that the intervention can be provided in a few minutes or via a brief leaflet. Interventions that can be delivered quickly and easily during clinical consultations and disseminated economically on a large scale are potentially particularly important.

Before embarking on the present trial, we piloted the 5:2 diet at our community weight loss clinics with inner city clients. The results suggested that the approach is likely to be attractive to such clients and have effects on par with those of standard weight management treatments. This initial anecdotal experience also identified two barriers to adherence to the 5:2 diet: The need to plan for the fasting days and have suitable foods prepared; and the hunger and discomfort on the fasting days. People who stuck with the programme for a few weeks seemed to have learned to cope with food provision requirements and to tolerate the fasting days better. Based on this experience, we hypothesised that effectiveness of the 5:2 diet will improve if dieters receive peer support to assist with adhering to the programme over the first few weeks.

The present trial had two aims. One was to assess long-term effects of simple 5:2 instructions compared to standard weight loss advice. Using simple instructions, typically from the internet, books or advice from friends, is how the diet is typically initiated. The second aim was to test the hypothesis derived from our pilot work, that encouraging adherence over the initial period improves long-term effects. To assess this, we included a study arm in which 5:2 dieters received weekly group support, to compare the results with simple 5:2 instructions.

## Materials and methods

### Study design and setting

This was a randomised controlled trial that had two aims. Firstly, to compare the effects of a standard weight loss advice on eating and exercise of the type typically provided within primary care, versus a simple 5:2 advice; and secondly, to compare the brief 5:2 advice with the same advice accompanied by weekly group support.

The trial took place in Tower Hamlets, an inner city area of high deprivation [32] at a Weight Management Clinic run by the Health and Lifestyle Research Unit, Queen Mary University of London. The clinic provides weight management services to the local community as an offshoot of its ongoing research programme.

Participants were recruited via leaflets and posters at GP practices and community venues and advertising in local newspapers and on social media.

### Participants

Participants were adults with a BMI ≥ 30 kg/m^2^ (or ≥28 kg/m^2^, with co-morbidities) aged 18 years and older who wanted to lose weight.

Exclusion criteria included pregnancy, breastfeeding, using insulin, history of eating disorders, currently taking medication prescribed by a psychiatrist, BMI>45 kg/m^2^, currently using the 5:2 diet, currently involved in other research, more than 5% body weight lost in the last 6 months, and unable to follow instructions and fill in forms in English.

### Procedures

Potential participants contacted the study team and were screened over the phone. If eligible, they were given an appointment and posted the Participant Information Sheet. Recruitment continued until the planned sample size was reached.

At the baseline visit, participants provided written informed consent and filled in baseline questionnaires. Participants were weighted and their blood pressure was measured. They were then randomised and received one of the three interventions delivered by research psychologists trained in study procedures. Follow-up data were collected face-to-face at six weeks and at six and twelve months, and over the phone at three months.

Participants received £20 to cover their travel and time at the 6 week and 6 month follow-up visits.

### Randomisation and blinding

Randomisation was computer-generated and cards labelled with a code representing the allocation were placed in opaque sealed envelopes. The list and envelopes were prepared by an independent researcher not involved in the study. Envelopes were held centrally and were opened in numerical sequence at the time of treatment assignment. Once the envelope was opened, the study allocation was documented by the study staff on the participant’s Clinical Record Form and the randomisation log. Staff conducting outcome assessments were blind to treatment allocation, until questions were reached that identified the study arm.

### Study arms

#### Standard Brief Advice (SBA)

Participants received a copy of the British Heart Foundation guides ‘Facts Not Fads’ and ‘Get Active, Stay Active’ plus the NHS ‘Change 4 Life’ series of booklets and a leaflet listing local resources for exercise. An advisor explained the programme, went over the key advice and tips in the written materials (e.g. portion control, food diaries, ‘eat-well’ plate, avoiding unnecessary snacks etc.), and answered questions. The individual session took approximately 20 minutes.

#### Self-help format of the 5:2 diet (5:2SH)

Participants received a leaflet on restricting their caloric intake to 500 kcal for women and 600 kcal for men, and on doing this on two non-consecutive days a week, with examples of meals containing the required amount of calories, and pointers to additional online support for 5:2 dieters. An advisor explained the programme and answered questions. Participants were also provided with the same leaflet about local resources for exercise that was given out with SBA. The individual session took approximately 20 minutes.

#### Group support format of the 5:2 diet (5:2G)

Participants received the 5:2SH intervention, but in addition were invited to attend six group support sessions (in weeks 1–6), each lasting one hour. Sessions were moderated by advisors. Participants were weighed and reported on their experience over the past week, whether they managed to adhere to the plan, whether they cook or use pre-prepared food on the fasting days, how they cope with hunger, etc. The focus of the sessions was on participants’ sharing their experience and maintaining motivation to carry on with 5:2. Participants were also encouraged to join an internet forum to report on their 5:2 adherence and weight change, and discuss their experience with other participants.

### Measures

The baseline questionnaire collected demographic details and history of attempts at weight loss. Objective measures included weight (using Omron bf400 scales), height and blood pressure (using Omron 705IT monitor).

Further questionnaire measures included the International Physical Activity Questionnaire (IPAQ) [33] (a questionnaire that includes 4 items to assess activity over the past 7 days) and Fat and Fibre Diet Behaviour Questionnaire (FFDBQ) [34] (a questionnaire with 31 items assessing diet content). IPAQ scores classified participants as engaging in low, moderate or high activity. FFDBQ provided scores for dietary intake of fat and fiber. These two questionnaires were included to see if there are any signs that 5:2 detracts dieters from other beneficial lifestyle changes.

Participants rated the interventions on four 10-point scales: Helpfulness of the intervention received (1 = not at all helpful to 10 = extremely helpful); difficulty in adhering to the intervention (1 = not at all difficult to 10 = extremely difficult); how likely they are to recommend the approach to others (1 = not at all likely to 10 = extremely likely); and readiness to continue (1 = not at all to 10 = completely).

Additionally, the SBA arm participants were asked whether they were using the tips provided in the intervention booklets and discussed with the advisor (Yes/No) and if Yes, which; while 5:2 arms participants reported number of fasting days adhered to over the previous time period and degree of hunger on fasting days (1 = not at all hungry; 10 = extremely hungry).

### Outcomes

The primary outcome was weight change in kg from baseline at six months. Secondary outcomes included weight change at other time points, percentage of participants who lost at least 5% of their baseline body weight at each time point, self-reported treatment adherence, ratings of the interventions, changes in FFDBQ (food choices) and IAPQ (exercise levels); use of other weight management methods; and changes in blood pressure.

### Sample size

In this early phase ‘first of its kind’ study, we opted for a pragmatic sample size achievable economically and quickly, but large enough to provide acceptable confidence intervals on the key estimates. We aimed to randomise 100 participants into each study arm. This sample size would provide 90% power (alpha 0.025 two-tailed) to detect a medium effect size (Cohen’s d between 0.5 and 0.7) when comparing change scores in the two relevant pairwise comparisons.

### Statistical analysis

Descriptive statistics were used for participants’ characteristics and measures of adherence. Continuous measures which met the parametric assumptions are described using the mean and standard deviation, while we report the median and interquartile range (IQR) when these assumptions were not met.

Analyses of weight change used the intention-to-treat (ITT) approach with the ‘last observation carried forward’ (LOCF) imputation for missing data, which is the most common approach in weight loss studies [35]. We also conducted a sensitivity analysis using the more conservative ‘baseline observation carried forward’ (BOCF) imputation. Although the assumption is often made that people gain weight over time, this does not seem to apply to people who are obese. A meta-analysis showed modest weight loss over time in people with BMI>30 [36]. BOCF is considered more conservative because participants tend to remain in the study while they are doing well and drop out when they relapse. Indeed, LOCF usually indicates a larger weight loss than BOCF [37]. We opted for conducting both analyses to allow comparisons with other studies.

For an additional sensitivity analysis, we used multiple imputation by chained equation to estimate missing data at each follow-up time point. In behaviour change studies that include direct contact with therapists, missingness is normally not random, as explained above, but multiple imputation is nevertheless sometimes recommended [38]. To compensate for the problem of missingness not being random, we included a number of auxiliary variables. We imputed outcomes 50 times, applying a set seed using the mi package in Stata with 50 dataset, and included in the imputation model baseline weight and auxiliary variables associated with each outcome and its missingness: Age, sex, education, employment status, ethnicity, number of previous attempts, greatest prior weight loss, systolic and diastolic blood pressure and whether participants qualify for free prescriptions. The estimated weight at follow-up was comparable for complete cases and various imputation methods (See S2 Table).

Differences in weight were analysed using linear regression with weight at follow-up regressed on study arm while adjusting for baseline weight. To compare the number of participants in different study arms who had achieved a weight loss of at least 5%, we used a binomial regression with a log link with weight loss status regressed on study arm. As the study focused on comparing SBA and 5:2G arms with 5:2SH, the 5:2SH arm was the reference group in the analyses.

Ratings of interventions are presented using median and IQR. Wilcoxon rank sum test was used to conduct the relevant pairwise comparisons (5:2SH vs SBA and 5:2SH vs. 5:2G) while adjusting for multiple testing. To assess whether the number of participants who used other weight management approaches differed between study arms, we used chi-square test or Fisher’s exact test if the cell size was small. To examine whether hunger levels changed over the six weekly ratings in the 5:2G arm, Friedman’s test was conducted using the participants with complete information.

Regarding blood pressure and reported fat intake, we compared scores between arms at 52 weeks while adjusting for baseline scores and estimated baseline adjusted means and mean differences for the two comparisons of interest. We also analysed change from baseline in fiber intake and activity levels (low, medium and high) at 52 weeks. For IPAQ, any reduction from medium or high activity was coded as decreased activity, while any increase from low or medium was coded as increased activity. Changes in fiber intake were coded as an increase or a decrease. Stata version 15 was used for data analyses.

As we conducted two pairwise comparisons (5:2 SH vs SB; and 5:2 SH vs 5:2G), we used a Bonferroni adjustment to account for multiple testing and reduce type I error (alpha = 0.05/2 = 0.025).

Ethical approval was provided by the London City Road and Hampstead Research Ethics Committee (16/LO/0073).

The trial was listed on the ISRCTN registry (ISRCTN79408248).

## Results

**Fig 1:**
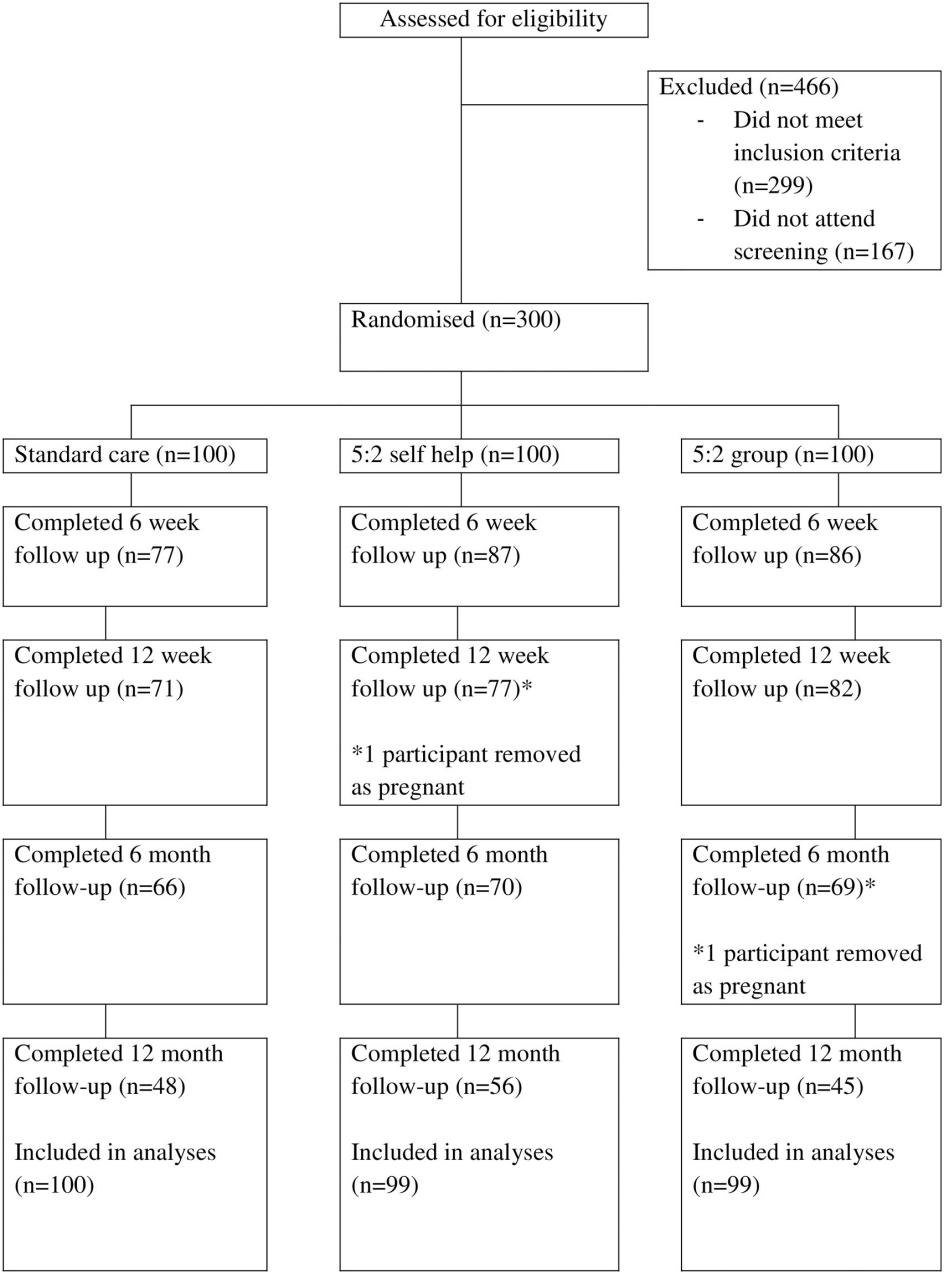
Consort diagram.

A total of 300 participants were recruited between 27 June 2016 and 15 June 2018, and followed up for one year. Fig 1 shows the participant flow through the trial. Two participants became pregnant during the follow-up period. Their data were included up to the point when pregnancy was reported, and excluded from analyses concerning weight changes afterwards. About half of the participants who started completed the study.

The median BMI of the sample was 33.8 kg/m^2^, 44% were entitled to free prescriptions, over half were from ethnic minorities, and two thirds were women. There were no significant differences between study arms in any of the baseline variables (Table 1).

**Table 1:** Sample characteristics.

	SBA	5:2 SH	5:2 G	Full sample
	(N = 95–100)*	(N = 95–100)*	(N = 94–100)*	(N = 284–300)*
Age Mean (SD)	47 (13)	51 (13)	47 (13)	48 (13)
Female N (%)	64 (64)	68 (68)	67 (67)	199 (66)
In paid employment N (%)	61 (61)	56 (57)	59 (59)	176 (59)
White British N (%)	42 (42)	46 (47)	46 (47)	134 (45)
White Other N (%)	17 (17)	10 (10)	9 (9)	36 (12)
Black N (%)	21 (21)	21 (21)	13 (13)	55 (19)
Asian N (%)	13 (13)	14 (14)	16 (16)	43 (14)
Mixed N (%)	4 (4)	6 (6)	9 (9)	19 (6)
Other N (%)	3 (3)	2 (2)	6 (6)	11 (4)
Receiving free prescriptions N (%)	39 (39)	50 (50)	44 (44)	133 (44)
Weight in kg Median (IQR)	95 (84–105)	92 (85–104)	95 (86–105)	95 (85–105)
BMI Median (IQR)	34.0 (30.7–37.7)	33.4 (31.7–37.7)	34.0 (31.7–37.7)	33.8 (31.1–37.5)
Largest past weight loss in kg Median (IQR)	12 (6–19)	11 (5–19)	11 (6–16)	11 (6–18)
Number of serious weight loss attempts Median (IQR)	3 (2–5)	3 (2–5)	3 (2–5)	3 (2–5)

*N varies due to missing data.

For continuous variables means (SD) are presented if parametric assumptions are met, otherwise we present the median (IQR).

Table 2 shows the weight change in kg in the three study arms using LOCF and BOCF analyses. The results of the multiple imputation analysis were similar (see S2 Table). Regarding the 5:2SH vs SBA comparison, both study arms achieved similar weight loss at six months and at one year. Regarding the 5:2SH vs 5:2G comparison, the initial advantage of 5:2G diminished over time. Residual plots indicated the assumption of homoscedasticity was met.

**Table 2:** Change in weight in kg compared to baseline (N = 100 in each study arm*).

	LOCF			BOCF		
	Mean (SD)	Differences (unadjusted)	Difference adjusted for baseline weight (95% CI), p	Mean (SD)	Differences (unadjusted)	Difference adjusted for baseline weight (95% CI), p
**6 weeks**						
SBA	-1.02 (2.5)	3.73 (-0.70–8.15)	0.56 (-0.70 to 1.19)	-1.0 (2.5)	3.73 (-0.70–8.15)	0.56 (-0.07 to 1.19)
			p = 0.081			p = 0.081
5:2 SH	-1.5 (2.1)	REF	REF	-1.5 (2.1)	REF	REF
5:2 G	-2.3 (2.1)	0.99 (-3.44–5.41)	-0.74 (-1.37 to -0.11)	-2.3 (2.1)	0.99 (-3.44–5.41)	-0.74 (-1.37 to -0.11)
			p = 0.02			p = 0.021
**24 weeks**						
SBA	-1.7 (4.4)	3.59 (-083- 8.00)	0.23 (-0.79 to 1.27)	-1.4 (4.4)	3.78 (-0.65–8.21)	0.42 (-0.60 to 1.45)
			p = 0.65			p = 0.42
5:2 SH	-1.8 (3.5)	REF	REF	-1.7 (3.5)	REF	REF
5:2 G	-2.3 (3.1)	1.46 (-2.97–5.88)	-0.43 (-1.46 to 0.60)	-1.9 (3.1)	1.76 (-2.67–6.20)	-0.13 (-1.15 to 0.90)
			p = 0.41			p = 0.81
**52 weeks**						
SBA	-1.8 (5.7)	3.55 (-0.98–8.08)	0.20 (-1.21 to 1.60)	-0.8 (4.5)	3.84 (-0.73–8.41)	0.41(-0.83to 1.64)
			p = 0.79			p = 0.52
5:2 SH	-1.9 (4.9)	REF	REF	-1.2 (4.5)	REF	REF
5:2 G	-2.6 (4.6)	1.25 (-3.39–5.79)	-0.64 (-2.04 to 0.77)	-1.1 (4.1)	2.07 (-2.51–6.65)	0.14 (-1.10 to 1.37)
			p = 0.37			p = 0.83

* One participant removed from 5:2 SH from 12 wks and one participant removed from 5:2 G from 26 wks when they became pregnant.

Table 3 shows the percentage of participants who lost at least 5% of their baseline weight. There were no significant differences between the three study arms.

**Table 3:** Percentage of participants losing at least 5% of their baseline body weight (N = 100 in each study arm*).

	LOCF		BOCF	
	N (%)	RR (95%CI), p	N (%)	RR (95%CI), p
**6 weeks**				
SBA	6 (6)	1.00 (0.33 to 3.0)	6 (6)	1.00 (0.33 to 3.00)
		p = 1.0		p = 1.00
5:2 SH	6 (6)	REF	6 (6)	REF
5:2 G	13 (13)	2.17 (0.86 to 5.47)	13 (13)	2.17 (0.86 to 5.47)
		p = 0.10		p = 0.10
**24 weeks**				
SBA	15 (15)	0.93 (0.49 to 1.77)	13 (13)	0.80 (0.41 to 1.58)
		p = 0.82		p = 0.53
5:2 SH	16 (16)	REF	16 (16)	REF
5:2 G	23 (23)	1.44 (0.81 to 2.55)	21 (21)	1.31 (0.73 to 2.36)
		p = 0.22		p = 0.36
**52 weeks**				
SBA	15 (15)	0.83 (0.44 to 1.54)	7 (7)	0.58 (0.24 to 1.41)
		p = 0.55		p = 0.23
5:2 SH	18 (18)	REF	12 (12)	REF
5:2 G	28 (28)	1.56 (0.92 to 2.62)	13 (13)	1.08 (0.52 to 2.26)
		p = 0.10		p = 0.83

* One participant removed from 5:2 SH from 12 wks and one participant removed from 5:2 G from 26 wks when they became pregnant.

Sensitivity analysis using multiple imputation led to the equivalent results (see S3 Table).

Retention rates, defined as the proportions of participants remaining in the study and providing study data, were similar in all three study arms. They were initially high, but gradually declined over time (see Table 4).

**Table 4:** Retention rates.

	SBA	5:2 SH	5:2 G
	(N = 100)	(N = 100)	(N = 100)
At 6 weeks N(%)	77 (77) *	87 (87) *	86 (86)
At 3 months N (%)	71 (71)	77 (77)	82 (82)
At 6 months N (%)	66 (66)	70 (70)	69 (69)
At 12 months N (%)	48 (48)	56 (56)	45 (45)

* N = 1 follow-up was completed over the phone.

The two 5:2 arms were asked at the 6 week follow up how many fast days they had completed since the start of the study (out of possible 14). Adherence in the 5:2G arm was higher. Counting non-responders as implementing 0 days, the 5:2SH arm reported a median of 10 (IQR = 6–12) fast days and 5:2G arm reported a median of 12 (IQR = 9–12) fast days (Mann-Whitney z = 2.24, p = .03).

Among 100 participants in the 5:2G arm, contact over support sessions 1 to 6 was maintained as follows: Session 1: 92 (all attended in person), Session 2: 89 (83 in person, 6 by phone), Session 3: 90 (74 in person, 16 by phone), Session 4: 90 (73 in person, 17 by phone), Session 5: 74 (64 in person, 10 by phone), and Session 6: 80 (71 in person, 9 by phone). In contrast to this, only six participants took up the offer to share their weekly progress via an online support forum, and those who did, posted information there only once or twice.

At the 6 week follow up, SBA arm participants were asked to list up to four tips from SBA advice and booklets that they were still using. Among 62 respondents, 60% reported changing their diet to more healthy food, 52% used portion control, 48% used exercise, 40% monitored their caloric intake e.g. via food diaries, 10% used ‘eat-well’ plate and 5% were avoiding unnecessary snacks. Ten other tips received one mention each.

The number of participants still following 5:2 at different time points decreased over time and did not differ between 5:2SH and 5:2G arms (6 weeks SH: N = 74 (74%) vs G: N = 81 (81%), chi2(1) = 1.41, p = 0.2; 24 weeks SH: N = 31 (31%) vs. G: N = 34 (34%); chi2(1) = 0.3, p = 0.62; 52 weeks SH: N = 22 (22%) vs. G: N = 17 (17%); chi2(1) = 0.8, p = 0.37).

In the 5:2G arm, 56 participants provided hunger ratings for each week. The severity of hunger on the fasting days decreased significantly over time (Friedman test chi^2^(4) = 40.9, p<0.001).

Ratings of the interventions were overall positive in all three study arms (see Table 5). However, 5:2SH intervention received significantly higher ratings for the likelihood of recommending the treatment to others and for readiness to continue than the SBA intervention; and 5:2G received more favourable ratings than 5:2SH regarding helpfulness and likelihood of recommending the treatment to others.

**Table 5:** Ratings of SBA and 5:2 interventions at week 6.

	SBA	5:2SH	5:2G	SBAvs. 5:2SH *	5:2G vs. 5:2SH *
	median (IQR)	median (IQR)	median (IQR)		
**How helpful did you find the intervention?**	7 (4–8)	8 (5–9)	10 (7–10)	Z = -1.6 p = 0.12	Z = -4.3 p<0.001
	(N = 75)	(N = 85)	(N = 85)		
**How likely is it that you will recommend it to others**	8 (5–10)	9 (7–10)	10 (9–10)	Z = -2.4 p = 0.02	Z = -3.7 p<0.001
	(N = 77)	(N = 87)	(N = 85)		
**Difficulty adhering to advice**	5 (3–7)	5 (1–7)	3 (2–5)	Z = 1.3 p = 0.20	Z = 1.1 p = 0.28
	(N = 73)	(N = 77)	(N = 82)		
**Readiness to continue**	8 (7–10)	10 (8–10)	10 (8–10)	Z = -3.3 p = 0.001	Z = 0.8 p = 0.43
	(N = 76)	(N = 77)	(N = 82)		

* Wilcoxon rank sum test.

### Effects of interventions on other outcomes

Participants in the SBA arm were more likely to try other weight management strategies than those in the two 5:2 arms over the first six months (see Table 6). The methods used included Slimming World, Weight Watchers, time-restricted eating, a variety of diets and meal replacements, Alli and Xenical. Some SBA participants also reported using the 5:2 diet (3, 4, 2 and 1 at 6W, 12W, 26W and 12M).

**Table 6:** Number (%) of participants reporting use of other weight management approaches at each time point.

	SBA	5:2 SH	5:2 G
**6 weeks (N = 249)**	9 (11.7)	4 (4.7)	2 (2.3)
**6 months (N = 202)**	15 (22.7)	6 (8.8)	7 (10.3)
**12months (N = 147)**	6 (12.8)	12 (21.4)	10 (22.7)
**Total N over 12 M** *	22 (26.2%)	19 (20.7%)	16 (18.2%)

* % calculated using the N of participants who responded at least once.

There was a small reduction in blood pressure and fat intake in all study arms, with no between-arms differences. Changes in physical activity and fiber intake were also comparable across the groups (see Table 7).

**Table 7:** Changes in blood pressure, fat intake, fiber intake and physical activity at 12 months.

	SBA	5:2 SH	5:2 G	SBA vs. 5:2 SH (mean difference 95%CI)	5:2G vs. 5:2 SH (mean difference, 95%CI)
**Adjusted Mean (SE)** *					
**Systolic BP**	-2.8 (2.4)	-4.7 (2.2)	-2.7	1.9 (-4.6–8.3)	1.0 (-4.6–8.6)
(N = 47,56, 44)					
**Diastolic BP**	-2.1 (1.5)	-1.3 (1.4)	-1.8 (1.6)	-0.78 (-4.9–3.3)	-0.53 (-4.7–3.7)
(N = 47,56, 44)					
**Fat intake**	-1.0 (0.30)	-0.7 (0.3)	-0.5 (0.3)	-0.3 (-1.0–0.5)	0.3 (-0.5–1.0)
(N = 28,32, 32)					
**N (%)**				RR (95%CI)	RR (95%CI)
**Fiber intake**					
(N = 45,53, 44)					
Increased	7 (15.6)	3 (5.7)	6 (13.6)	1.14 (0.42–3.13)	0.42 (0.11–1.56)
Decreased	11 (24.4)	7 (13.2)	11 (25.0)	0.98 (0.47–2.02)	0.53 (0.22–1.25)
No change	27 (60.0)	43 (81.1)	27 (61.4)	0.98 (0.70–1.37)	1.32 (1.01–1.73)
**IPAQ**					
(N = 46,51, 43)					
Increased	15 (32.6)	15 (310)	14 (32.6)	1.00 (0.55–1.82)	0.92 (0.50–1.68)
Decreased	12 (26.1)	8 (16.0)	8 (18.6)	1.40(0.64–3.10)	0.86(0.35–2.10)
No change	19 (41.3)	27 (54.0)	21 (48.8)	0.85 (0.53–1.34)	0.74–1.65)

* The adjusted means are adjusted for baseline scores.

## Discussion

A simple 5:2 intervention obtained better user ratings than standard brief advice, but long-term weight loss in the two study arms was similar. Group support improved 5:2 results in terms of weight loss over the initial 6 weeks, but the difference was no longer significant at 6 and 12 months. At one year, 15%, 18% and 28% of participants lost at least 5% of their body weight in the SBA, 5:2SH and 5:2G study arms, respectively. The 5:2 intervention had no adverse effects on activity levels or healthy eating.

The main strengths of the trial are that it provided the first information on the effectiveness of the simple 5:2 diet advice, and that it evaluated the method in ‘real-life’ setting. The main limitation is that although the trial was larger than most previous studies of intermittent fasting, some findings of borderline significance could have become clearer if the sample size was larger. SBA arm participants were also more likely to report using alternative treatments. It would not be ethical (or practical) to try to stop participants from trying alternative methods, but this could have in theory contributed to masking an intervention effect. We conducted an exploratory sensitivity analysis excluding participants using non-allocated methods and while there was a small increase in some differences between the study arms, results remained in line with the primary analysis (see S4 Table).

As with previous studies of IER [26–28], we detected little difference between the efficacy of the 5:2 and the active control condition. Although the 5:2 diet approach is expected to be more ‘forgiving’ than most other IER approaches, adherence to 5:2 declined from some 80% at 6 weeks to some 20% at one year. This tallies with a recent study that reported adherence to a dietitian-supervised 5:2 diet declining to 21% in week 50. In that study, more participants stopped practicing their intervention with 5:2 than with continuous energy restriction between years 1 and 2, although weight loss was similar in both conditions [34]. The limited caloric allowance on the fasting days seems to generate a degree of hunger and discomfort that proves eventually too severe for most clients.

The caloric allowance on fasting days is based on an estimated 25% of mean self-reported daily caloric intake of some 2,000 calories per day for women and 2,400 for men. However, objectively assessed intakes suggest that the norms for the UK population are in fact over 3,000 calories for men and some 2,400 for women [39, 40]. It is possible that increasing the 5:2 allowances to about a third of average daily caloric intake, i.e. 800 calories for women and 1,000 calories for men, could improve adherence and balance the slower weight loss with higher participant retention. Alternatively, other more lenient and simple IER approaches, such as time-restricted eating, may have a better potential for long-term adherence by a higher proportion of clients who try them.

Group support in the 5:2G arm generated a greater weight loss initially. Disappointingly, the online forum, which was scheduled to provide ongoing support, was hardly used. Future studies should explore ways of providing on-going support by other means, such as via telephone support or texting [41].

In participants who persevered throughout the initial few weeks, there was a significant reduction of hunger ratings. This suggests that persevering with the programme leads to habituation to the discomfort that the diet initially generates, and/or participants learn to cope with the discomfort more effectively. The finding justifies an advice that clinician and clients may find useful, i.e. a reassurance that, provided dieters continue with the programme, the initial discomfort is likely to subside.

The 5:2 diet did not detract from any positive behaviour changes following conventional advice, but no study arm implemented any significant changes. The finding is nevertheless reassuring in suggesting that IER interventions may not undermine the standard advice on healthy lifestyle.

The implications of the finding that brief one-off 5:2 instructions achieved the same result as the standard weight loss advice can be seen in two different ways. One is that the results are only modest, and so neither intervention is worthwhile. However, it is also possible to argue that the one-year weight loss of at least 5% of the baseline body weight in 15%-18% of participants is not negligible; and that the 5:2 advice, which received more favourable user ratings, is also much simpler and easier to deliver than multimodal briefing on several complex lifestyle changes. From this perspective, health professionals who provide brief weight management advice may consider including the 5:2 option.

In the UK, GPs currently have an option to refer patients to free intensive specialist weight management treatments. Such a referral can be short and represents the gold standard for brief GP intervention [41]. Most patients referred to such treatments however do not attend them [41]. Even within the UK system, and more so in countries that do not have such provisions, a suggestion to try 5:2 could be provided in a quick consultation and be useful especially for patients who had not benefitted from the standard advice.

## Conclusions

In summary, a simple explanation of the 5:2 diet generated similar modest long-term outcomes as the traditional more complex advice and written instructions concerning diet and exercise. 5:2 accompanied by group support generated better early outcomes, but the effect weakened over time. Future studies should consider higher caloric allowances on the fast days, and a provision of ongoing support. Clinicians providing brief advice on weight management may consider recommending the 5:2 diet. The approach is not superior to the standard multimodal advice, but it is simpler and more attractive to users.

## Supporting information

S1 Table Missing weight outcomes at each time point. (DOCX)

S2 Table Mean (SD) of weight in kg in complete cases and imputed data. (DOCX)

S3 Table Regression results with missing data estimated using multiple imputation. (DOCX)

S4 Table Exploratory sensitivity analysis excluding participants using non-allocated treatments. (DOCX)

S1 File CONSORT 2010 checklist of information to include when reporting a randomised trial*. (DOC)

S2 File Protocol. (DOCX)

S3 File 5–2 CRF. (DOC)

S4 File 5–2 Self-help CRF. (DOC)

S5 File 5–2 Standard advice CRF. (DOC)

S6 File Calorie leaflet. (DOCX)

S7 File Exercise handout 5–2 study. (DOC)

S8 File 5–2 guide. (DOCX)

S9 File Dataset. (SAV)

## References

[1] Bluher M. Obesity: global epidemiology and pathogenesis. Nat Rev Endocrinol. 2019;15(5):288–98. doi: 10.1038/s41574-019-0176-8 30814686

[2] Zaninotto P, Head J, Stamatakis E, Wardle H, Mindell J. Trends in obesity among adults in England from 1993 to 2004 by age and social class and projections of prevalence to 2012. Journal of Epidemiology & Community Health. 2009;63(2):140–6. doi: 10.1136/jech.2008.077305 19074182

[3] Ayala-Marín AM, Iguacel I, Miguel-Etayo PD, Moreno LA. Consideration of Social Disadvantages for Understanding and Preventing Obesity in Children. Frontiers in Public Health. 2020;8:423. doi: 10.3389/fpubh.2020.00423 32984237 PMC7485391

[4] Poobalan AS, Aucott LS, Smith WC, Avenell A, Jung R, Broom J. Long-term weight loss effects on all cause mortality in overweight/obese populations. Obes Rev. 2007;86):503–13. doi: 10.1111/j.1467-789X.2007.00393.x 17949355

[5] Brown A, Leeds A. Very low-energy and low-energy formula diets: Effects on weight loss, obesity co-morbidities and type 2 diabetes remission–an update on the evidence for their use in clinical practice. Nutrition bulletin. 2019;44(1):7–24.

[6] Lean ME, Leslie WS, Barnes AC, Brosnahan N, Thom G, McCombie L, et al. Primary care-led weight management for remission of type 2 diabetes (DiRECT): an open-label, cluster-randomised trial. The Lancet. 2018;391(10120):541–51. doi: 10.1016/S0140-6736(17)33102-1 29221645

[7] Hall KD, Kahan S. Maintenance of lost weight and long-term management of obesity. Medical Clinics. 2018;102(1):183–97. doi: 10.1016/j.mcna.2017.08.012 29156185 PMC5764193

[8] BBC. Horizon: Eat, Fast and Live Longer. Screened on BBC Two on 6 August 2012.

[9] Mosley M, Spencer M. The fast diet. London: Short Books; 2013.

[10] Fisher R. What is the 5:2 diet?: BBC Good Food; [16/9/2019]. https://www.bbcgoodfood.com/howto/guide/what-52-diet.

[11] Wing RR, Epstein LH, Marcus MD, Koeske R. Intermittent low-calorie regimen and booster sessions in the treatment of obesity. Behaviour research and therapy. 1984;22(4):445–9. doi: 10.1016/0005-7967(84)90086-x 6477369

[12] Varady K. Intermittent versus daily calorie restriction: which diet regimen is more effective for weight loss? Obesity reviews. 2011;12(7):e593–e601. doi: 10.1111/j.1467-789X.2011.00873.x 21410865

[13] Brown JE, Mosley M, Aldred S. Intermittent fasting: a dietary intervention for prevention of diabetes and cardiovascular disease? The British Journal of Diabetes & Vascular Disease. 2013;13(2):68–72.

[14] Varady KA, Bhutani S, Church EC, Klempel MC. Short-term modified alternate-day fasting: a novel dietary strategy for weight loss and cardioprotection in obese adults. The American journal of clinical nutrition. 2009;90(5):1138–43. doi: 10.3945/ajcn.2009.28380 19793855

[15] Arguin H, Dionne IJ, Sénéchal M, Bouchard DR, Carpentier AC, Ardilouze J-L, et al. Short-and long-term effects of continuous versus intermittent restrictive diet approaches on body composition and the metabolic profile in overweight and obese postmenopausal women: a pilot study. Menopause. 2012;19(8):870–6. doi: 10.1097/gme.0b013e318250a287 22735163

[16] Antoni R, Johnston KL, Collins AL, Robertson MD. Intermittent v. continuous energy restriction: differential effects on postprandial glucose and lipid metabolism following matched weight loss in overweight/obese participants. British Journal of Nutrition. 2018;119(5):507–16. doi: 10.1017/S0007114517003890 29508693

[17] Harvie M, Wright C, Pegington M, McMullan D, Mitchell E, Martin B, et al. The effect of intermittent energy and carbohydrate restriction v. daily energy restriction on weight loss and metabolic disease risk markers in overweight women. British Journal of Nutrition. 2013;110(8):1534–47. doi: 10.1017/S0007114513000792 23591120 PMC5857384

[18] Harvie MN, Pegington M, Mattson MP, Frystyk J, Dillon B, Evans G, et al. The effects of intermittent or continuous energy restriction on weight loss and metabolic disease risk markers: a randomized trial in young overweight women. International journal of obesity. 2011;35(5):714–27. doi: 10.1038/ijo.2010.171 20921964 PMC3017674

[19] Klempel MC, Kroeger CM, Bhutani S, Trepanowski JF, Varady KA. Intermittent fasting combined with calorie restriction is effective for weight loss and cardio-protection in obese women. Nutrition journal. 2012;11(1):98. doi: 10.1186/1475-2891-11-98 23171320 PMC3511220

[20] Kroeger CM, Klempel MC, Bhutani S, Trepanowski JF, Tangney CC, Varady KA. Improvement in coronary heart disease risk factors during an intermittent fasting/calorie restriction regimen: Relationship to adipokine modulations. Nutrition & metabolism. 2012;9(1):98. doi: 10.1186/1743-7075-9-98 23113919 PMC3514278

[21] Klempel MC, Bhutani S, Fitzgibbon M, Freels S, Varady KA. Dietary and physical activity adaptations to alternate day modified fasting: implications for optimal weight loss. Nutrition journal. 2010;9(1):35. doi: 10.1186/1475-2891-9-35 20815899 PMC2941474

[22] Klempel MC, Kroeger CM, Varady KA. Alternate day fasting (ADF) with a high-fat diet produces similar weight loss and cardio-protection as ADF with a low-fat diet. Metabolism. 2013;62(1):137–43. doi: 10.1016/j.metabol.2012.07.002 22889512

[23] Eshghinia S, Mohammadzadeh F. The effects of modified alternate-day fasting diet on weight loss and CAD risk factors in overweight and obese women. Journal of Diabetes & Metabolic Disorders. 2013;12(1):4. doi: 10.1186/2251-6581-12-4 23497604 PMC3598220

[24] Williams KV, Mullen ML, Kelley DE, Wing RR. The effect of short periods of caloric restriction on weight loss and glycemic control in type 2 diabetes. Diabetes care. 1998;21(1):2–8. doi: 10.2337/diacare.21.1.2 9538962

[25] Hill JO, Schlundt DG, Sbrocco T, Sharp T, Pope-Cordle J, Stetson B, et al. Evaluation of an alternating-calorie diet with and without exercise in the treatment of obesity. The American journal of clinical nutrition. 1989;50(2):248–54. doi: 10.1093/ajcn/50.2.248 2667313

[26] Trepanowski JF, Kroeger CM, Barnosky A, Klempel MC, Bhutani S, Hoddy KK, et al. Effect of alternate-day fasting on weight loss, weight maintenance, and cardioprotection among metabolically healthy obese adults: a randomized clinical trial. JAMA internal medicine. 2017;177(7):930–8. doi: 10.1001/jamainternmed.2017.0936 28459931 PMC5680777

[27] Headland ML, Clifton PM, Keogh JB. Effect of intermittent compared to continuous energy restriction on weight loss and weight maintenance after 12 months in healthy overweight or obese adults. International journal of obesity. 2019;43(10):2028–36. doi: 10.1038/s41366-018-0247-2 30470804

[28] He S, Wang J, Zhang J, Xu J. Intermittent Versus Continuous Energy Restriction for Weight Loss and Metabolic Improvement: A Meta-Analysis and Systematic Review. Obesity. 2021;29(1):108–15. doi: 10.1002/oby.23023 34494373

[29] Harris L, Hamilton S, Azevedo LB, Olajide J, De Brún C, Waller G, et al. Intermittent fasting interventions for treatment of overweight and obesity in adults: a systematic review and meta-analysis. JBI Evidence Synthesis. 2018;16(2):507–47. doi: 10.11124/JBISRIR-2016-003248 29419624

[30] Welton S, Minty R, O’Driscoll T, Willms H, Poirier D, Madden S, et al. Intermittent fasting and weight loss. Systematic review. 2020;66(2):117–25. 32060194 PMC7021351

[31] Davis C, Clarke R, Coulter S, Rounsefell K, Walker R, Rauch C, et al. Intermittent energy restriction and weight loss: a systematic review. European journal of clinical nutrition. 2016;70(3):292–9. doi: 10.1038/ejcn.2015.195 26603882

[32] https://www.towerhamlets.gov.uk/Documents/Borough_statistics/Income_poverty_and_welfare/Indices_of_Deprivation_Low_resolution.pdf.

[33] Craig CL, Marshall AL, Sjöström M, Bauman AE, Booth ML, Ainsworth BE, et al. International physical activity questionnaire: 12-country reliability and validity. Medicine & science in sports & exercise. 2003;35(8):1381–95. doi: 10.1249/01.MSS.0000078924.61453.FB 12900694

[34] Shannon J, Kristal AR, Curry SJ, Beresford S. Application of a behavioral approach to measuring dietary change: the fat-and fiber-related diet behavior questionnaire. Cancer Epidemiology and Prevention Biomarkers. 1997;6(5):355–61. 9149896

[35] Elobeid MA, Padilla MA, McVie T, Thomas O, Brock DW, Musser B, et al. Missing data in randomized clinical trials for weight loss: scope of the problem, state of the field, and performance of statistical methods. PloS one. 2009;4(8):e6624. doi: 10.1371/journal.pone.0006624 19675667 PMC2720539

[36] Whitlock G, Lewington S, Sherliker P, Clarke R, Emberson J, Halsey J, et al. Body-mass index and cause-specific mortality in 900 000 adults: collaborative analyses of 57 prospective studies. Lancet. 2009;373(9669):1083–96. doi: 10.1016/S0140-6736(09)60318-4 19299006 PMC2662372

[37] Jørgensen AW, Lundstrøm LH, Wetterslev J, Astrup A, Gøtzsche PC. Comparison of results from different imputation techniques for missing data from an anti-obesity drug trial. PLoS One. 2014;9(11):e111964. doi: 10.1371/journal.pone.0111964 25409438 PMC4237333

[38] Batterham MJ, Tapsell LC, Charlton KE. Analyzing weight loss intervention studies with missing data: which methods should be used? Nutrition. 2013;29(7–8):1024–9. doi: 10.1016/j.nut.2013.01.017 23644010

[39] Harper H, Hallsworth M. Counting Calories. How under-reporting can explain the apparent fall in calorie intake The Behavioural Insights Team. 2016. 10.1038/sj.bdj.2016.639 27608574

[40] Office for National Statistics. A Government Statistical Service perspective on official estimates of calorie consumption. Office for National Statistics; 2016.

[41] Lewis E, Huang H-CC, Hassmén P, Welvaert M, Pumpa KL. Adding Telephone and Text Support to an Obesity Management Program Improves Behavioral Adherence and Clinical Outcomes. A Randomized Controlled Crossover Trial. International Journal of Behavioral Medicine. 2019;26(6):580–90. doi: 10.1007/s12529-019-09815-1 31512155

