# Supplementary material for: A randomised controlled trial of the 5:2 diet
Source: PLoS One. 2021 Nov 17;16(11):e0258853. doi: 10.1371/journal.pone.0258853 (PMC8598045; doi:10.1371/journal.pone.0258853)
Supplement: S1 Table — (DOCX) [file pone.0258853.s001.docx]

**S1 Table. Missing weight outcomes at each time point.**

|  | **SBA**  **(N =100)** | **5:2 SH**  **(N =100)** | **5:2 G**  **(N =100)** |
| --- | --- | --- | --- |
| Weight at 6 weeks | 23 (23) | 13 (13) | 14 (14) |
| Weight at 12 weeks | 40 (40) | 35 (35) | 26 (26) |
| Weight at 24 weeks | 35 (35) | 30 (30) | 31 (31) |
| Weight at 52 weeks | 53 (53) | 44 (44) | 56 (56) |
