# Supplementary material for: A randomised controlled trial of the 5:2 diet
Source: PLoS One. 2021 Nov 17;16(11):e0258853. doi: 10.1371/journal.pone.0258853 (PMC8598045; doi:10.1371/journal.pone.0258853)
Supplement: S2 Table — (DOCX) [file pone.0258853.s002.docx]

**S2 Table. Mean (SD) of weight in kg in complete cases and imputed data.**

|  | **SBA** | **5:2 SH** | **5:2 G** |
| --- | --- | --- | --- |
| **Weight at 6 weeks** |  |  |  |
| Completers  (N=77 -87-86) | 95.7 (17.9) | 92.6 (14.2) | 93.8 (15.7) |
| LOCF | 97.0 (17.7) | 93.2 (14.5) | 94.1 (15.3) |
| BOCF | 97.0 (17.7) | 93.2 (14.5) | 94.2 (15.3) |
| MI* | 96.5 (17.4) | 93.1 (14.3) | 93.9 (15.2) |
| **Weight at 12 weeks** |  |  |  |
| Completers  (N =60 -65-74) | 95.6 (19.4) | 92.4 (14.5) | 91.4 (16.4) |
| LOCF | 96.1 (17.5) | 91.8 (13.8) | 92.7 (15.5) |
| BOCF | 96.2 (17.5) | 92.0 (13.9) | 93.1 (15.7) |
| MI* | 95.1 (17.3) | 91.3 (13.8) | 92.2 (15.3) |
| **Weight at 24 weeks** |  |  |  |
| Completers  (N = 65 -70-69) | 95.4 (16.8) | 92.8 (14.9) | 93.0 (15.9) |
| LOCF | 96.3 (17.2) | 92.7 (14.3) | 94.1 (15.8) |
| BOCF | 96.6 (17.4) | 92.8 (14.2) | 94.6 (15.9) |
| MI* | 95.9 (16.9) | 92.8 (14.3) | 93.8 (15.7) |
| **Weight at 52 weeks** |  |  |  |
| Completers  (N=47 -56-44) | 94.9 (16.3) | 93.0 (16.5) | 96.1 (10.0) |
| LOCF | 96.1 (17.0) | 92.6 (14.9) | 93.8 (16.8) |
| BOCF | 97.2 (17.3) | 93.3 (15.2) | 95.4 (16.5) |
| MI* | 95.8 (17.0) | 92.8 (15.1) | 93.9 (16.9) |
| **Weight change**  **(BL-52Wks)** |  |  |  |
| Completers  (N=47 -56-44) | 1.8 (6.5) | 2.1 (5.9) | 2.4 (6.0) |
| LOCF | 1.8 (5.7) | 1.9 (4.8) | 2.6 (4.6) |
| BOCF | 0.8 (4.5) | 1.2 (4.5) | 1.1 (4.1) |
| MI* | 2.2 (7,6) | 1.9 (6.3) | 2.6 (6.1) |

*Average score of 50 imputed dataset.
