# Supplementary material for: A randomised controlled trial of the 5:2 diet
Source: PLoS One. 2021 Nov 17;16(11):e0258853. doi: 10.1371/journal.pone.0258853 (PMC8598045; doi:10.1371/journal.pone.0258853)
Supplement: S3 Table — (DOCX) [file pone.0258853.s003.docx]

**S3 Table. Regression results with missing data estimated using multiple imputation.**

|  | **Weight loss since baseline^^^** | **Loss of ≥5% of baseline body weight^#^** |
| --- | --- | --- |
|  | b (95% CI), p | RR (95% CI), p |
| **6 weeks** |  |  |
| SBA | 0.3 (-0.5 to 1.0), p=0.48 | 1.2 (0.4 to 3.5), p=0.76 |
| 5:2 SH | REF | REF |
| 5:2 G | -0.9 (-1.6- -0.2), p=0.02 | 2.0 (0.8- 5.0), p=0.12 |
| **12 weeks** |  |  |
| SBA | 0.5 (-0.6 to 1.6), p=0.35 | 0.8 (0.5 to 1.4), p=0.54 |
| 5:2 SH* | REF | REF |
| 5:2 G | -1.0 (-2.0- 0.04), p=0.06 | 1.5 (1.0- 2.3), p=0.05 |
| **24 weeks** |  |  |
| SBA | 0.1 (-1.3 to 1.3), p=0.95 | 1.0 (0.6 to 1.9), p=0.91 |
| 5:2 SH* | REF | REF |
| 5:2 G** | -0.7 (-2.0- 0.6), p=0.29 | 1.5 (0.9- 2.5), p=0.15 |
| **52 weeks** |  |  |
| SBA | -0.1 (-2.2 to 2.1), p=0.96 | 1.0 (0.6- 1.7) p=0.95 |
| 5:2 SH* | REF | REF |
| 5:2 G** | -0.6 (-2.7- 1.5), p=0.59 | 1.3 (0.8- 2.2), p=0.32 |

* One participant removed from 5:2 SH from 12 wks as pregnant and information was not imputed

** One participant removed from 5:2 G from 26 wks as pregnant and information was not imputed

^ Linear regression regressing follow-up weight onto study arm while adjusting for baseline weight

# Log-binomial regression regressing weight loss status (>5% loss: yes/no) onto study arm
