# Supplementary material for: A randomised controlled trial of the 5:2 diet
Source: PLoS One. 2021 Nov 17;16(11):e0258853. doi: 10.1371/journal.pone.0258853 (PMC8598045; doi:10.1371/journal.pone.0258853)
Supplement: S4 Table — (DOCX) [file pone.0258853.s004.docx]

**S4 Table. Exploratory sensitivity analysis excluding participants using non-allocated treatments**

**Table 4a.** Sensitivity analysis excluding participants who were using alternative weight management approaches

| LOCF | | | | BOCF | | |
| --- | --- | --- | --- | --- | --- | --- |
|  | Mean (SD) | Difference | Difference adjusted for baseline weight (95% CI), p | Mean (SD) | Difference | Difference adjusted for baseline weight (95% CI), p |
| **6 weeks** |  |  |  |  |  |  |
| SBA  N = 91 | -0.8 (2.2) | 3.67 (-0.91- 8.25) | 0.69 (-0.07 to 1.31)  p=0.03 | -0.8  (2.2) | 3.67 (-0.91- 8.25) | 0.69 (-0.07 to 1.31)  p=0.03 |
| 5:2 SH  N = 96 | -1.5 (2.1) | REF | REF | -1.5 (2.1) | REF | REF |
| 5:2 G  N = 98 | -2.3 (2.1) | 0.56 (-3.94- 5.06) | -0.83 (-1.43 to -0.22)  p=0.01 | -2.3 (2.1) | 0.56 (-3.94- 5.06) | -0.83 (-1.43 to -0.22)  p=0.01 |
| **24 weeks** |  |  |  |  |  |  |
| SBA  N = 81 | -1.3 (4.3) | 3.06 (-1.70- 7.83) | 0.42 (-0.61 to 1.45)  p=0.43 | -0.9 (4.2) | 3.29 (-1.49- 8.07) | 0.63 (-0.39 to 1.64)  p=0.23 |
| 5:2 SH  N = 89 | -1.6 (3.0)* | REF | REF | -1.5 (2.9)* | REF | REF |
| 5:2 G  N = 91 | -2.4 (3.0)** | 1.27 (-3.35- 5.90) | -0.68 (-1.68 to 0.32)  p=0.18 | -1.9 (3.0)** | 1.60 (-3.04- 6.24) | -0.36 (-1.34 to 0.63)  p=0.48 |
| **52 weeks** |  |  |  |  |  |  |
| SBA  N = 78 | -1.1 (4.3) | 2.58 (-2.42- 7.59) | 0.69 (-0.62 to 2.00)  p=0.30 | -0.4 (2.7) | 2.24 (-2.82- 7.30) | 0.30 (-0.78 to 1.37)  p=0.59 |
| 5:2 SH  N = 80 | -1.7 (4.3) * | REF | REF | -0.7 (3.8) * | REF | REF |
| 5:2 G  N = 83 | -3.0 (4.0) ** | -0.72 (-5.65- 4.21) | -1.24 (-2.53 to 0.04)  p=0.06 | -1.1 (3.6) ** | 0.13 (-4.85- 5.11) | -0.41 (-1.46 to 0.65)  p=0.45 |

* One participant removed from 5:2 SH from 12 wks as pregnant

** One participant removed from 5:2 G from 26 wks as pregnant

**Table 4b.** Sensitivity per protocol analysis of the percentage of participants losing at least 5% of their baseline body weight treating participants using alternative weight management approaches as not successful.

| LOCF | | | BOCF | |
| --- | --- | --- | --- | --- |
| ***At least 5% reduction in weight*** | | | | |
|  | N (%) | RR (95%CI), p | N (%) | RR (95%CI), p |
| **6 weeks** |  |  |  |  |
| SBA | 4 (4) | 0.8 (0.22 to 2.89)  p=0.73 | 4 (4) | 0.8 (0.22 to 2.89)  p=0.73 |
| 5:2 SH | 5 (5) | REF | 5 (5) | REF |
| 5:2 G | 13 (13) | 2.6 (0.96 to 7.02)  p=0.06 | 13 (13) | 2.6 (0.96 to 7.02)  p=0.06 |
| **24 weeks** |  |  |  |  |
| SBA | 9 (9) | 0.7 (0.33 to 1.68)  p=0.48 | 7 (7) | 0.58 (0.24 to 1.41)  p=0.23 |
| 5:2 SH | 12 (12) ^*^ | REF | 12 (12)^*^ | REF |
| 5:2 G | 21 (21) ^**^ | 1.8 (0.91 to 3.36)  p=0.09 | 19 (19) ^**^ | 1.58 (0.81 to 3.08)  p=0.18 |
| **52 weeks** |  |  |  |  |
| SBA | 9 (9) | 0.69 (0.31 to 1.53)  p=0.36 | 5 (5) | 0.71 (0.23 to 1.15)  p=0.54 |
| 5:2 SH | 13 (13) ^*^ | REF | 7 (7)^*^ | REF |
| 5:2 G | 25 (25) ^**^ | 1.92 (1.05 to 3.54)  p=0.04 | 10 (10)^**^ | 1.43 (0.57 to 3.60)  p=0.45 |

* One participant removed from 5:2 SH from 12 wks as pregnant

** One participant removed from 5:2 G from 26 wks as pregnant
