# Supplementary material for: A randomised controlled trial of the 5:2 diet
Source: PLoS One. 2021 Nov 17;16(11):e0258853. doi: 10.1371/journal.pone.0258853 (PMC8598045; doi:10.1371/journal.pone.0258853)
Supplement: S2 File — (DOCX) [file pone.0258853.s006.docx]

1. **TITLE PAGE**

**Full Title** Short-term effects of standard weight management advice and of the 5-2 diet delivered in self-help or group support format

**Short Title/Acronym 5-2 Study**

**Sponsor** Queen Mary, University of London

*Contact person of the above sponsor organisations is:*

Dr Sally Burtles

Queen Mary University of London

Joint Research Management Office

Queen Mary Innovation Centre

5 Walden Street

London

E1 2EF

Phone: 020 7882 7260

Email: [sponsorsrep@bartshealth.nhs.uk](mailto:sponsorsrep@bartshealth.nhs.uk)

**REC Reference 16/LO/0073**

**Chief Investigator** Professor Peter Hajek

Health and Lifestyle Research Unit

2 Stayner’s Road

London

E1 4AH

**Site** Health and Lifestyle Research Unit

2 Stayner’s Road

London

E1 4AH

**Funder** Medical Research Council (MRC)

**Contents Page**

Table of Contents

2. GLOSSARY of Terms and Abbreviations 3

3. SIGNATURE PAGE 4

4. SUMMARY/SYNOPSIS 5

5. INTRODUCTION 6

6. TRIAL OBJECTIVES 8

7. METHODOLOGY 8

8. STUDY PROCEDURES 10

9. STATISTICAL CONSIDERATIONS 13

10. ETHICS 14

11. DATA HANDLING AND RECORD KEEPING: 14

12. PRODUCTS, DEVICES, TECHNIQUES AND TOOLS 15

13. MONITORING & AUDITING 15

14. SAFETY REPORTING 15

15. TRIAL COMMITTEES 15

16. FINANCE AND FUNDING 15

17. INDEMNITY 15

18. DISSEMINATION OF RESEARCH FINDINGS 16

19. REFERENCES 16

### ****GLOSSARY of Terms and Abbreviations****

AE Adverse Event

CI Chief Investigator

CRF Case Report Form

ICF Informed Consent Form

IPAQ International Physical Activity Questionnaire
JRMO Joint Research Management Office

Participant An individual who takes part in a clinical trial

PIS Participant Information Sheet

R&D Research and Development

RCT Randomised Controlled Trial

REC Research Ethics Committee

TSC Trial Steering Committee

### SIGNATURE PAGE

**Chief Investigator Agreement**

The clinical study as detailed within this research protocol **(Version 2.6, dated 26 May 2017)**, or any subsequent amendments will be conducted in accordance with the Research Governance Framework for Health & Social Care (2005), the World Medical Association Declaration of Helsinki (1996) and the current applicable regulatory requirements and any subsequent amendments of the appropriate regulations.

**Chief Investigator Name:** Professor Peter Hajek

**Chief Investigator Site:** Health and Lifestyle Research Unit, 2 Stayner’s Road, London E1 4AH

**Signature and Date:** P.HAJEK 26 May 2017

### SUMMARY/SYNOPSIS

| **Short Title** | 5-2 Study |
| --- | --- |
| **Methodology** | Randomised controlled trial |
| **Study Centre** | Health and Lifestyle Research Unit, 2 Stayner’s Road, E1 4AH |
| **Objectives/Aims** | 1. Determine the effectiveness of the 5-2 diet combined with multi-session group support compared to the 5-2 diet delivered in a self-help format and to standard advice on diet and physical activity, in terms of weight loss at 6 weeks, 3 months (self-reported), 6 and 12 months 2. Explore predictors of, and barriers and facilitators to, adherence to the 5-2 diet. |
| **Number of Participants** | 300 |
| **Main Inclusion Criteria** | Adults, aged 18 years and older, with a BMI ≥30kg/m2, or BMI≥28kg/m2 with comorbidities |
| **Statistical Methodology and Analysis (if applicable)** | The primary analysis will compare weight loss between the three groups at 6 months, adjusting for baseline weight. We propose to implement a strict intention to treat (ITT) analysis as our primary outcome, with participants lost to follow-up included as achieving no weight loss. |
| **Study Duration** | 30 months |

### INTRODUCTION

Over a quarter of adults in England are obese [1], with obesity more prevalent in disadvantaged socioeconomic groups [2, 3]. Obesity is associated with a number of adverse health conditions including cardiovascular disease, type 2 diabetes, osteoarthritis and a number of cancers. Weight loss has been shown to improve many of these illnesses, and to reduce all-cause mortality [4, 5], with a maintained weight loss of 5% being sufficient to produce clinically significant benefits [6].

Treatments for obesity are an important focus across health research, policy, and commissioning arenas. Frontline health professionals are expected to assist people with weight problems by recommending healthy eating and physical activity, complemented as needed by drug therapy and a referral to a local weight management service. Local weight management services now come under the remit of public health departments at local councils. Public health commissioners typically contract non-NHS providers of varied quality. The services typically offer advice on food and physical activity combined with varied types of behavioural support. As with other services that provide face-to-face lifestyle interventions, only a small minority of the target group accesses them. The results in terms of throughput and weight loss are not generally monitored and so remain largely unknown.

Substantial resources are now being invested in such services and there is an urgent need to identify approaches with the best reach and with at least some evidence base.

**Intermittent Fasting and the 5-2 diet**

A novel type of intermittent energy restriction (IER) was introduced in the UK by a BBC Horizon documentary screened in August 2012 [7]. The ‘Fast Diet’ [8] or ‘5-2 Diet’ has since attracted much attention among people trying to lose weight, and it remains highly popular. The simple principle of this approach is caloric restriction (500 kcal for women and 600 kcal for men) on two non-consecutive days each week [8].

IER is not a new concept. The first RCT of IER was published some 30 years ago [9]. However, the previous versions of IER required fasting on every other day or on consecutive days, and they also required caloric restrictions on non-fasting days. Unsurprisingly, they did not have a wide appeal, but several studies have been published.

Narrative reviews of IER [13–15] draw heavily on animal data and highlight the paucity of human trials, but we identified several studies evaluating earlier versions of IER [9–12, 16–23]. Typically, participants were asked to restrict energy by 75% every other day or on two consecutive days per week, sometimes after a period of very-low calorie diet, and they were also asked to use prescriptive diets on non-fasting days. IER was as effective as continuous energy restriction and had better effects on e.g. insulin sensitivity and lipid profiles. This provides encouraging proof-of-principle evidence, but the generalizability of the results and practicability of these interventions is limited. The studies were small and participants were carefully selected. In some studies they had to pass a pre-test of 2-day energy restriction and typically over 50% of eligible participants declined participation or were unable to cope with the pre-test fasting.

The main advantage of the 5-2 diet responsible for its popularity seems to be its simplicity, the recommendation that fasting takes place on separate days (‘going hungry’ for two consecutive days seems too difficult for most people), and the removal of food restrictions on non-fasting days. Unlike most other approaches to weight loss, the 5-2 diet does not require relentless self-control, but allows people to stop worrying about food intake on 5 days a week. Numerous internet fora provide extensive reports of success and document considerable public enthusiasm for the 5-2 diet. Compared to previous approaches to intermittent fasting, this less restrictive regime may have a weaker effect, but this is likely to be compensated for by better adherence and sustainability of the intervention. The approach is easy to disseminate and given its attractiveness to dieters, if it is effective, it could have a considerable potential for wide-spread use and for public health benefit.

No trials have evaluated this version of IER so far and there is a need to evaluate its short-term effects when delivered in the formats amenable for wide-spread use and with populations that will most benefit from such options.

**Risks and benefits**

The 5-2 diet generates a degree of hunger and discomfort on the fasting days, especially initially, but apart from this it conveys no known risks. The potential benefits include weight loss [15], improvements in insulin levels and insulin sensitivity [24, 25], and possible improvements in circulating cholesterol, triglycerides, and blood pressure [26, 27].

**Rationale for the current study**

Given the current popularity of the 5-2 diet, and the lack of data on its effectiveness, information is needed on whether it generates short-term weight loss and what is the most effective mode of its delivery, particularly in the community settings where standard approaches have low penetration and low efficacy.

A number of clients at our weight management programmes have reported their experience with the 5-2 diet and we have also piloted its use at our clinics. This identified some barriers such as finding suitable foods and planning meals for the fasting days, but the main hurdle to implementing the 5-2 diet is the hunger and discomfort on the fasting days. Our experience confirms that people who stick with the programme typically learn to tolerate the fasting days well, especially once they see the positive results. We now believe that although the diet in its usual self-help format can help some people, it is likely to be more effective if group support is included to assist adherence over the first few weeks. After this initial period, the diet should be much easier to maintain without further external support.

We propose to investigate the initial effects of the 5-2 diet delivered in two different formats (self-help and with group support), both of which are amenable for wide-spread use.

### TRIAL OBJECTIVES

To evaluate the short-term weight loss achieved with the 5-2 diet combined with multi-session behavioural support compared to the 5-2 diet delivered in a self help format and standard advice on diet and physical activity.

**Primary Outcome**

Weight loss at six months

**Secondary Outcomes**

1. Changes in weight, blood pressure, dietary habits, exercise levels and proportion of participants losing at least 5% of their body weight, at 6 weeks, 6 and 12 months and the self-reported change in weight at 3 months
2. Adherence to the three interventions, determinants of adherence, and barriers and facilitators to adherence.

**Trial Design**

Randomised controlled trial (RCT)

### METHODOLOGY

**Inclusion Criteria**

- Age 18 years and older
- BMI≥30kg/m2, or BMI≥28kg/m2 with comorbidities

**Exclusion criteria**

- Women who are pregnant, breastfeeding or planning to conceive in the next 6 months
- History of eating disorders
- Currently involved in another research project
- Currently using 5:2 diet
- Non English speakers
- Unavailable for 6 month follow-up
- BMI>45kg/m2
- Lost more than 5% of their body weight in the last 6 months
- Taking medication prescribed by a psychiatrist (these medications can affect weight and psychiatric illness makes adherence to long-term programs difficult)
- Diabetics who require insulin
- Participant has undergone any form of bariatric surgery

We are not excluding any other co-morbidities to ensure the study addresses NHS needs and the results are generalizable.

**Criteria for premature withdrawal**

Participants will be followed up at 12 months unless they explicitly want no further contact.

**Study Design / Plan – Study Visits**

**Study Scheme Diagram**

| **Recruitment**  Potential participants will contact the study team via phone/email after seeing study advertisements in local newspapers and on the internet. Staff will provide information about the study and if the potential participant is interested, they will be screened. If eligible, they will receive an appointment for the baseline visit and sent the Participant Information Sheet (PIS) and booking confirmation. The phone call will take up to 20 minutes. | | | | | | | |
| --- | --- | --- | --- | --- | --- | --- | --- |
|  |  | | |  | |  |  |
| **Baseline visit**  Participants will receive information about the study and provide written informed consent; baseline data will be obtained including weight, height, blood pressure and demographic details; eating and physical activity questionnaires will be completed.  Participants will be randomised to 5-2 Behavioural support, 5-2 self-help, or standard advice arms. | | | | | | | |
|  | |  |  | |  | |  |
| **5-2 self-help (5-2 SH)**  **(N=100)**  20 minute session on the 5-2 plan, a written guide on how to use it with recommendations for internet and other resources and a leaflet listing local resources for exercise. | |  | **5-2 plus group support (5-2 GS) (N=100)**  Participants receive the same materials as in the 5-2 self-help condition and attend 6 weekly group support sessions. | |  | **Standard advice self help (SH) (N=100)**  20-minute session  on healthy eating and physical activity and the British Heart Foundation guides Facts Not Fads and Get Active, Stay Active plus the NHS Change  4 Life series of booklets and a leaflet listing local resources for exercise. | |
|  | |  |  | |  |  | |
| **6 week follow up**  Measurement of weight and blood pressure; recording of any concomitant weight management treatments; adherence to and ratings of 5-2 diet | |  | **6 week follow up**  Measurement of weight and blood pressure; recording of any concomitant weight management treatments; adherence to and ratings of 5-2 diet | |  | **6 week follow up**  Measurement of weight and blood pressure; recording of any concomitant weight management treatments; adherence to and ratings of advice components | |
|  | |  |  | |  |  | |
| **3 month follow up phone call**  Self-report weight; adherence to and ratings of 5-2 diet; recording of any concomitant weight management treatments | |  | **3 month follow up phone call**  Self-report weight; adherence to and ratings of 5-2 diet; recording of any concomitant weight management treatments | |  | **3 month follow up phone call**  Self-report weight; adherence to and ratings of the advice components; recording of any concomitant weight management treatments | |

| **6 month follow up**  Measurement of weight and blood pressure; adherence to and ratings of 5-2 diet; recording of any concomitant weight management treatments; change in eating habits; change in exercise levels (IPAQ) |  | **6 month follow up**  Measurement of weight and blood pressure; adherence to and ratings of 5-2 diet; recording of any concomitant weight management treatments; change in eating habits; change in exercise levels (IPAQ) |  | **6 month follow up**  Measurement of weight and blood pressure; adherence to and ratings of the advice components; recording of any concomitant weight management treatments; change in eating habits; change in exercise levels (IPAQ) |
| --- | --- | --- | --- | --- |

Pending their consent to continue in the study, participants will be followed up at 12-months (see the above 6 month follow-up for details of assessment).

### STUDY PROCEDURES

**Recruitment**

Participants would be recruited via the following methods; advertising in newspapers including the Evening Standard, Metro and local papers; adverts on social media and on internet fora and through GP practices; online bulletins; leaflets and posters in community venues; leaflets via mail drops; texting previous weight clinic participants.

**Informed Consent**

Participants will receive a PIS with details of the study and will have at least 24 hours before signing the informed consent form (ICF).

**Screening**

Participants who meet the inclusion criteria over the phone will attend the baseline session. They will provide written informed consent, provide baseline measures and get randomised to one of the three study arms.

**Randomisation**

Randomisation would be computer-generated by the study statistician, using envelopes to reveal the randomisation allocation.

**Schedule of Assessment**

| **Time** | **Baseline** | **6 weeks** | **3 months** | **6 and 12 months** |
| --- | --- | --- | --- | --- |
| Demographics | X |  |  |  |
| Weight loss history | X |  |  |  |
| Weight | X | X | X (self-report) | X |
| Height | X |  |  |  |
| Blood pressure | X | X |  | X |
| Co-morbidities | X | X | - | X |
| Concurrent medications | X | X | - | X |
| International Physical Activity Questionnaire (IPAQ) | X | X | - | X |
| Diet Quality Questionnaire | X | X | - | X |
| Use of non-trial slimming methods | X | X | X | X |
| Feedback on treatment |  | X | - | X |
| Adherence to 5-2/advice |  | X* | X* | X* |
| Ratings of 5-2/advice |  | X** | - | X** |

**Measures**

- Baseline questionnaire: Age, gender, ethnicity, education, employment, health status, weight history and history of attempts at weight loss
- Weight, height and blood pressure
- International Physical Activity Questionnaire (IPAQ)[28]: The IPAQ measures frequency, duration of time spent on walking, vigorous, moderate intensity and sedentary activity over the past 7 days
- The Fat- and Fiber-related Diet Behaviour Questionnaire (FFDBQ) [29]: The HEI is a measure of diet quality based on the U.S. dietary guidelines and includes both food and nutrient-based indicators.
- *Adherence to 5-2: Number of fasting days adhered to; calories consumed per fast day
- *Adherence to standard advice: Changes in eating and exercise levels
- **Ratings of 5-2/standard advice: Difficulty of adhering to 5:2 plan/changes in eating habits (1=not at all difficult; 10=extremely difficult); degree of hunger on fasting days (5:2 arms only) (1=not at all hungry; 10=extremely hungry), readiness to continue with the fast days/changes in eating and exercise (1=not at all; 10=completely ready); helpfulness of the 5-2/standard advice (1=not at all; 10=extremely); Whether they would recommend 5-2/standard advice to others (1=not at all likely; 10=extremely likely)

**End of Study Definition**

The last participant completes the 12-month follow-up.

**Participant withdrawal**

Participants will be able to withdraw from the study at any time. Data already collected will be used in the final analysis, provided the participant consents to their data collected to date to be used.

**Study arm treatments**

**5-2 self-help (5-2 SH) arm** would receive a 20-minute session on the 5-2 plan and a written guide on how to use it with recommendations for internet resources along with a leaflet listing local resources for exercise. Participants will be encouraged to purchase and use pedometers to increase their exercise levels and to assess their weight weekly. In both 5-2 arms, participants will be asked to reduce their energy intake on two non-consecutive days per week to 500 calories for women and 600 calories for men. Participants will be seen by Research Health Psychologists.

**5-2 plus group support (5-2 GS) arm** would receive a brief explanation of the 5-2 plan and will be booked for six weekly group support sessions. Participants would also receive a leaflet listing local resources for exercise and will be encouraged to purchase and use pedometers to increase their exercise levels and to assess their weight weekly. The sessions will focus on helping clients to adhere to 5-2, share coping and fasting strategies and maintain motivation. Each session would last for approximately one hour and groups will comprise of 10-20 participants. Research Health Psychologists will run the groups.

**Standard advice self-help (SH) arm** would receive a 20-minutes session with an advisor focusing on healthy eating and physical activity and instructions on using the ‘Facts not Fads’ and the ‘Get Active, Stay Active’ British Heart Foundation self-help guides, supplemented by the NHS Change 4 Life series of booklets and a leaflet listing local resources for exercise. Participants will be encouraged to use pedometers to increase their exercise levels and to assess their weight weekly. Participants will be seen by Research Health Psychologists.

All sessions will be held at the Health and Lifestyle Research Unit, 2 Stepney Green, London E1 4AH.

All participants will receive £20 for their travel and time at the 6 week and 6 and 12 month follow-up visits.

**Data collection and follow-up for withdrawn subjects**

Participants will be followed up at 6 weeks, 3 months (over the phone) and 6 and 12 months unless they explicitly want no further contact.

### STATISTICAL CONSIDERATIONS

**Primary Endpoint Efficacy Analysis**

Change in weight from baseline at 6 months after enrolment into the study.

**Secondary Endpoint Efficacy Analysis**

Secondary analyses will also be conducted at 6 weeks and 3 and 12 months months after enrolment into the study.

**Safety Endpoints**

There are no safety endpoints for this trial.

**Statistical Analysis**

Analyses will be undertaken after the last participant has completed the 6-month follow-up. ITT analysis will be conducted as the primary outcome, with participants lost to follow-up included as achieving no weight loss. 'Last-weight-carried-forward' imputation and 'completers only' analysis limited to participants who provide follow-up data are reported as sole outcomes in many weight loss trials. To allow comparison across studies, we will conduct such analyses as well.

Weight change will be analysed by analysis of variance (ANOVA) with comparisons between all pairs of the three groups.

We shall also examine the effect of age, socioeconomic grouping, BMI at baseline and gender on weight loss and include any univariately significant variables in an analysis of covariance (ANCOVA) of weight change alongside study arm allocation. We shall assess, via interaction, the moderator effects of these variables on weight loss.

We will also calculate the proportion of randomised participants who achieved at least 5% weight loss and odds ratios for percentage weight change will be analysed via logistic regression.

Further to this, we will report group sessions attended in the 5-2 BS arm, and adherence to 5-2 in both 5-2 arms, together with 5-2 ratings; and report adherence to and evaluation of elements of the advice provided in SH arm, concerning changes in diet and exercise levels. IPAQ and FFDBQ scores in the three study arms will be compared using ANOVA.

**Sample Size**

In this early phase primary research, we opted for a pragmatic sample size

achievable economically and quickly but large enough to provide reasonable confidence intervals on the key estimates. We aim to randomise 100 participants in each study arm.

### ETHICS

This protocol and any subsequent amendments, along with any accompanying material provided to the patient in addition to any advertising material will be submitted by the Investigator to an Independent Research Ethics Committee (REC). Written Approval from the Committee must be obtained and subsequently submitted to the JRMO to obtain Final R&D approval.

There are no conflicts of interest to report.

### DATA HANDLING AND RECORD KEEPING:

**Confidentiality**

Participants data will remain confidential and will be handled, processed, stored and destroyed according to the terms of the Data Protection Act. We will not request any medical information about participants from their doctors. Copies of all documents relevant to the study will be kept in the trial master file at the Health and Lifestyle Research Unit, 2 Stayner’s Road, London E1 4AH.

Only study staff and representatives of the sponsor or regulatory authorities will have potential access to view study data that could be linked to patient identifiable data. All data on the CRFs and in the database will be pseudonymised and not contain any identifying information.

**Study documents**

- A signed protocol and any subsequent amendments
- Current/superseded Patient Information Sheets
- Current/superseded Consent Forms
- Indemnity documentation from sponsor
- Conditions of Sponsorship from sponsor
- Conditional/Final R&D approval
- Ethics submissions/approvals/correspondence
- CVs of CI and staff
- Good Clinical Practice (GCP) certificates of CI and staff
- Delegation log
- Patient identification log
- Screening log
- Enrolment log

**Registration and Case Report Form**

**Registration form**

Participant demographic and baseline details will be collected using the standard treatment form. These will be kept separately from the CRF.

**Case Report Form**

The elements of the CRF include:

- Eligibility/exclusion criteria checklist
- Visit details
- Study questionnaires

**Record retention and Archiving**

During the course of research, study records will be kept in secure conditions. When the study is complete, records will be kept for a further 20 years as per QMUL standard protocol.

### PRODUCTS, DEVICES, TECHNIQUES AND TOOLS

We will use the Omron HBF400 Body Fat Monitor and Scale and the Omron Blood Pressure Monitor with Interface (705IT).

### MONITORING & AUDITING

No audits are required. Sponsor will follow own SOPs for monitoring and auditing.

### SAFETY REPORTING

Neither standard weight loss advice nor 5:2 are expected to generate adverse events, but we will monitor the health status of participants at each follow-up.

### TRIAL COMMITTEES

The trial steering committee will consist of PI, study manager, and trial statistician.

### FINANCE AND FUNDING

This study is funded by the Medical Research Council (MRC).

### INDEMNITY

The JRMO has arranged for suitable indemnity concerning negligent harm to be in place for this study. Indemnity will be provided by Queen Mary, University of London.

The insurance that Queen Mary, University of London has in place provides “No Fault Compensation” for participants which provides an indemnity to participants for non-negligent harm.

### DISSEMINATION OF RESEARCH FINDINGS

Study results will be published in a peer-reviewed journal and may be presented at scientific conferences.

### REFERENCES

1. Health and Social Care Information Centre: Statistics on Obesity, Physical Activity and Diet - England, 2014. standard; 2014.

2. Stamatakis E, Wardle J, Cole TJ: **Childhood obesity and overweight prevalence trends in England: evidence for growing socioeconomic disparities**. Int J Obes 2005 2010, **34**:41–47.

3. Zaninotto P, Head J, Stamatakis E, Wardle H, Mindell J: **Trends in obesity among adults in England from 1993 to 2004 by age and social class and projections of prevalence to 2012**. J Epidemiol Community Health 2009, **63**:140–146.

4. Avenell A, Broom J, Brown TJ, Poobalan A, Aucott L, Stearns SC, Smith WCS, Jung RT, Campbell MK, Grant AM: **Systematic review of the long-term effects and economic consequences of treatments for obesity and implications for health improvement**. Health Technol Assess Winch Engl 2004, **8**:iii–iv, 1–182.

5. Poobalan AS, Aucott LS, Smith WCS, Avenell A, Jung R, Broom J: **Long-term weight loss effects on all cause mortality in overweight/obese populations**. Obes Rev Off J Int Assoc Study Obes 2007, **8**:503–513.

6. Blackburn G: Effect of degree of weight loss on health benefits. *Obes Res* 1995, 3 Suppl 2:211s–216s.

7. **BBC Two - Horizon, 2012-2013, Eat, Fast and Live Longer** [http://www.bbc.co.uk/programmes/b01lxyzc]

8. Mosley DM, Spencer M: *The Fast Diet: The Secret of Intermittent Fasting - Lose Weight, Stay Healthy, Live Longer*. London: Short Books; 2013.

9. Wing RR, Epstein LH, Marcus MD, Koeske R: **Intermittent low-calorie regimen and booster sessions in the treatment of obesity**. *Behav Res Ther* 1984, **22**:445–449.

10. Arguin H, Dionne IJ, Sénéchal M, Bouchard DR, Carpentier AC, Ardilouze J-L, Tremblay A, Leblanc C, Brochu M: **Short- and long-term effects of continuous versus intermittent restrictive diet approaches on body composition and the metabolic profile in overweight and obese postmenopausal women: a pilot study**. *Menopause N Y N* 2012, **19**:870–876.

11. Klempel MC, Kroeger CM, Bhutani S, Trepanowski JF, Varady KA: **Intermittent fasting combined with calorie restriction is effective for weight loss and cardio-protection in obese women**. *Nutr J* 2012, **11**:98.

12. Kroeger CM, Klempel MC, Bhutani S, Trepanowski JF, Tangney CC, Varady KA: **Improvement in coronary heart disease risk factors during an intermittent fasting/calorie restriction regimen: Relationship to adipokine modulations**. *Nutr Metab* 2012, **9**:98.

13. Varady KA: **Intermittent versus daily calorie restriction: which diet regimen is more effective for weight loss?: Weight loss by calorie restriction diets**. *Obes Rev* 2011, **12**:e593–e601.

14. Varady KA, Bhutani S, Church EC, Klempel MC: **Short-term modified alternate-day fasting: a novel dietary strategy for weight loss and cardioprotection in obese adults**. *Am J Clin Nutr* 2009, **90**:1138–1143.

15. Brown JE, Mosley M, Aldred S: **Intermittent fasting: a dietary intervention for prevention of diabetes and cardiovascular disease?** *Br J Diabetes Vasc Dis* 2013, **13**:68–72.

16. Klempel MC, Bhutani S, Fitzgibbon M, Freels S, Varady KA: **Dietary and physical activity adaptations to alternate day modified fasting: implications for optimal weight loss**. *Nutr J* 2010, **9**:35.

17. Klempel MC, Kroeger CM, Varady KA: **Alternate day fasting (ADF) with a high-fat diet produces similar weight loss and cardio-protection as ADF with a low-fat diet**. *Metabolism* 2013, **62**:137–143.

18. Eshghinia S, Mohammadzadeh F: **The effects of modified alternate-day fasting diet on weight loss and CAD risk factors in overweight and obese women**. *J Diabetes Metab Disord* 2013, **12**:4.

19. Harvie MN, Pegington M, Mattson MP, Frystyk J, Dillon B, Evans G, Cuzick J, Jebb SA, Martin B, Cutler RG, Son TG, Maudsley S, Carlson OD, Egan JM, Flyvbjerg A, Howell A: **The effects of intermittent or continuous energy restriction on weight loss and metabolic disease risk markers: a randomized trial in young overweight women**. *Int J Obes* 2011, **35**:714–727.

20. Harvie M, Wright C, Pegington M, McMullan D, Mitchell E, Martin B, Cutler RG, Evans G, Whiteside S, Maudsley S, Camandola S, Wang R, Carlson OD, Egan JM, Mattson MP, Howell A: **The effect of intermittent energy and carbohydrate restriction v. daily energy restriction on weight loss and metabolic disease risk markers in overweight women**. *Br J Nutr* 2013, **110**:1534–1547.

21. Williams KV, Mullen ML, Kelley DE, Wing RR: **The effect of short periods of caloric restriction on weight loss and glycemic control in type 2 diabetes**. *Diabetes Care* 1998, **21**:2–8.

22. Hill JO, Schlundt DG, Sbrocco T, Sharp T, Pope-Cordle J, Stetson B, Kaler M, Heim C: **Evaluation of an alternating-calorie diet with and without exercise in the treatment of obesity**. *Am J Clin Nutr* 1989, **50**:248–254.

23. Ash S, Reeves MM, Yeo S, Morrison G, Carey D, Capra S: **Effect of intensive dietetic interventions on weight and glycaemic control in overweight men with Type II diabetes: a randomised trial**. *Int J Obes Relat Metab Disord J Int Assoc Study Obes* 2003, **27**:797–802.

24. Halberg N, Henriksen M, Söderhamn N, Stallknecht B, Ploug T, Schjerling P, Dela F: **Effect of intermittent fasting and refeeding on insulin action in healthy men**. *J Appl Physiol Bethesda Md 1985* 2005, **99**:2128–2136.

25. Tikoo K, Tripathi DN, Kabra DG, Sharma V, Gaikwad AB: **Intermittent fasting prevents the progression of type I diabetic nephropathy in rats and changes the expression of Sir2 and p53**. *FEBS Lett* 2007, **581**:1071–1078.

26. Katare RG, Kakinuma Y, Arikawa M, Yamasaki F, Sato T: **Chronic intermittent fasting improves the survival following large myocardial ischemia by activation of BDNF/VEGF/PI3K signaling pathway**. *J Mol Cell Cardiol* 2009, **46**:405–412.

27. Nomani MZ, Hallak MH, Nomani S, Siddiqui IP: **Changes in blood urea and glucose and their association with energy-containing nutrients in men on hypocaloric diets during Ramadan fasting**. *Am J Clin Nutr* 1989, **49**:1141–1145.

28. Craig CL, Marshall AL, Sjöström M, *et al.* **International physical activity questionnaire: 12-country reliability and validity.** *Med Sci Sports Exerc* 2003;**35**:1381–95.

29. Shannon J, Kristal AR, Curry SJ, Beresford SA. **Application of a Behavioural Approach to Measuring Dietary Change: The Fat- and Fiber-related Diet Behaviour Questionnaire.** Cancer Epidemiol. Biomarkers Prev. 1997, 6:335-361.
