# Supplementary material for: A randomised controlled trial of the 5:2 diet
Source: PLoS One. 2021 Nov 17;16(11):e0258853. doi: 10.1371/journal.pone.0258853 (PMC8598045; doi:10.1371/journal.pone.0258853)
Supplement: S4 File — (DOC) [file pone.0258853.s008.doc]

CLINICAL RECORD FORM

MRC 5-2 Study

**5-2 *–* 6-week follow-up**

Today’s date_____________ Weight ________ kg Blood pressure _______________ mmHg

Are you currently using any ‘non-study’ weight control methods, products or medications? Yes No

**If yes**, what are you using? _________________________________________________________

Over the last 6 weeks, how many fast days did you complete? ____

Are you still on the 5-2 plan? Yes No

**If No,** what was the main reason you stopped? __________________________________

**If Yes,** please complete the questions below:

On average, how many calories do you consume on a fast day? ______ calories

How ready are you to continue with the fast days?: (1=not at all to 10=completely ready) ______

How difficult is it to stick to 5:2 these days? (1=not at all difficult to 10= extremely difficult) ____

How hungry are you on the fast days? (1=not at all hungry to 10=extremely hungry) _____

What type of food do you use on the fast days? Pre-packaged Home cooked Both

Compared to before you started the 5-2 diet, are the fast days now:

**Easier More difficult The same difficulty** to complete (circle one)

How helpful did you find the 5-2 guide? (1=not at all helpful to 10=extremely helpful) ____

How likely would you be to recommend the 5-2 guide to others? (1=not at all likely to 10=extremely likely) _______

On your fast days, is there a part of the day when you feel more hungry or uncomfortable than at

other times of the day?:

| MORNING | AFTERNOON | EVENING | AT NIGHT | NO CLEAR DIFFERENCE |
| --- | --- | --- | --- | --- |

Do you have any other feedback on the 5-2 guide? If yes, please provide details:

______________________________________________________________________________

**On your 5-2 days:**

Please mark with ticks the hours when you typically eat/drink (eating anything at all, however small, counts, including drinks that contain sugar). If you eat/drink sugar drinks outside the times shown, please add appropriate boxes

| 7am | 8 | 9 | 10 | 11 | 12 | 1pm | 2 | 3 | 4 | 5 | 6 | 7 | 8 | 9 | 10 | 11 | 12am |
| --- | --- | --- | --- | --- | --- | --- | --- | --- | --- | --- | --- | --- | --- | --- | --- | --- | --- |
|  |  |  |  |  |  |  |  |  |  |  |  |  |  |  |  |  |  |

**On your *normal* (non-5:2) weekday:**

Please mark with ticks the hours when you typically eat/drink (eating anything at all, however small, counts, including drinks that contain sugar). If you eat/drink sugar drinks outside the times shown, please add appropriate boxes

| 7am | 8 | 9 | 10 | 11 | 12 | 1pm | 2 | 3 | 4 | 5 | 6 | 7 | 8 | 9 | 10 | 11 | 12am |
| --- | --- | --- | --- | --- | --- | --- | --- | --- | --- | --- | --- | --- | --- | --- | --- | --- | --- |
|  |  |  |  |  |  |  |  |  |  |  |  |  |  |  |  |  |  |

**At the first session, we provided a list of exercise options in your local area. We are trying to find out whether such lists are useful.**

Did you contact any provider from the list?:  Yes   No

**If Yes, which one?** ___________________________

Did you attend any sessions with them? Yes No   **If Yes, how many?:** _______________

Do you have any other feedback regarding the leaflet or the provider?:

___________________________________________________________________________

Have there been any changes to your health or medications since you joined the study? Yes No

**If Yes,** please list them here: ________________________________________________________

**INTERNATIONAL PHYSICAL ACTIVITY QUESTIONNAIRE**

The questions below ask about the **last 7 days**. Please think about the activities you do at work, at home, to get from

place to place, and in your spare time for recreation, exercise or sport.

1. Think about all the **vigorous** activities that you did in the **last 7 days**. This refers to activities that take hard

physical effort and make you breathe much harder than normal. Think *only* about those physical activities that

you did for at least 10 minutes at a time.

a) During the **last 7 days**, on how many days did you do **vigorous** physical activities like heavy lifting, digging, aerobics, or fast cycling?

_____ **days per week**

No vigorous physical activities ***Skip to question 2***

How much time did you usually spend doing **vigorous** physical activities on one of those days?

_____ **hours per day** _____ **minutes per day** Don’t know/Not sure

1. Now, think about all the **moderate** activities that you did in the **last 7 days**. **Moderate** activities refer to activities

that take moderate effort and make you breathe somewhat harder than normal. Think only about those physical

activities that you did for at least 10 minutes at a time.

a) During the **last 7 days,** on how many days did you do **moderate** physical activities like carrying light loads,

cycling at a regular pace or doubles tennis? Do not include walking.

_____ **days per week**

No moderate physical activities ***Skip to question 3***

How much time did you usually spend doing **moderate** physical activities on one of those days?

_____ **hours per day** _____ **minutes per day** Don’t know/Not sure

1. Now, think about the time you spent **walking** in the **last 7 days**. This includes at work and at home, walking to

travel from place to place, and any other walking that you might do solely for recreation, sport, exercise, or leisure.

a) During the **last 7 days**, on how many days did you **walk** for at least 10 minutes at a time?

_____ **days per week**

No walking ***Skip to question 4***

b) How much time did you usually spend **walking** on one of those days?

_____ **hours per day** _____ **minutes per day** Don’t know/Not sure

1. The last question is about the time you spent **sitting** on weekdays during the **last 7 days**. Include time spent

at work, at home, while doing course work and during leisure time. This may include time spent sitting at a desk,

visiting friends, reading, or sitting or lying down to watch television.

During the **last 7 days**, how much time did you spend **sitting** on a **week day**?

_____ **hours per day** _____ **minutes per day** Don’t know/Not sure

**FAT AND FIBER QUESTIONNAIRE**

For each of the questions below, please tick **one** response that most applies to you

| In the past 6 weeks how often did you… | | | | |
| --- | --- | --- | --- | --- |
|  | Usually or always | Often | Sometimes | Rarely or never |
| Eat frozen yoghurt or sorbet instead of ice cream? |  |  |  |  |
| Use low-calorie salad dressing instead of regular? |  |  |  |  |
| Eat low-fat cheese instead of regular cheese? |  |  |  |  |
| Drink skimmed or semi-skimmed milk instead of whole? |  |  |  |  |
| Use low-fat spray oil instead of oil, margarine or butter? |  |  |  |  |
| Eat a potato without butter or margarine? |  |  |  |  |
| Eat bread with butter or margarine? |  |  |  |  |
| Put butter or margarine on vegetables? |  |  |  |  |
| Take the skin off chicken? |  |  |  |  |
| Eat baked or boiled chicken? |  |  |  |  |
| Trim visible fat from your meat? |  |  |  |  |
| Eat a small portion of meat? (e.g. the size of a deck of cards) |  |  |  |  |
| Eat baked or broiled fish? |  |  |  |  |
| Eat raw vegetables for a snack? |  |  |  |  |
| Eat two or more vegetables at dinner? |  |  |  |  |
| Eat a vegetable at lunch? |  |  |  |  |
| Eat fruit for dessert? |  |  |  |  |
| Eat a vegetarian dinner? |  |  |  |  |
| Eat meatless pasta sauce? |  |  |  |  |
| Eat fish or chicken instead of red meat? |  |  |  |  |
| Eat high-fiber cereals? e.g bran flakes |  |  |  |  |
| Eat cereal (hot or cold) for breakfast? |  |  |  |  |
| Eat whole-grain crackers or bread? |  |  |  |  |
| Add bran to casseroles or cereal? |  |  |  |  |
| Eat raw vegetables for snacks instead of crisps? |  |  |  |  |
| Eat fruit for breakfast? |  |  |  |  |
| Eat whole-wheat instead of regular pasta? |  |  |  |  |
| Eat brown rice instead of white rice? |  |  |  |  |

**5-2 *–* 12-week follow-up**

Today’s date_____________ Self reported weight ________ kg

Are you currently using any ‘non-study’ weight control methods, products or medications? Yes No

**If yes**, what are you using? _________________________________________________________

How many weeks did you complete the 5-2 for? ____

Are you still on the 5-2 plan? Yes No

**If No,** what was the main reason you stopped? __________________________________

**If Yes,** please complete the questions below:

On average, how many calories do you consume on a fast day? ______ calories

How ready are you to continue with the fast days?: (1=not at all to 10=completely ready) ______

How difficult is it to stick to 5:2 these days? (1=not at all difficult to 10= extremely difficult) ____

How hungry are you on the fast days? (1=not at all hungry to 10=extremely hungry) _____

What type of food do you use on the fast days? Pre-packaged Home cooked Both

Compared to before you started the 5-2 diet, are the fast days now:

**Easier More difficult The same difficulty** to complete (circle one)

On your fast days, is there a part of the day when you feel more hungry or uncomfortable than at

other times of the day?:

| MORNING | AFTERNOON | EVENING | AT NIGHT | NO CLEAR DIFFERENCE |
| --- | --- | --- | --- | --- |

**On your 5-2 days:**

Please mark with ticks the hours when you typically eat/drink (eating anything at all, however small, counts, including drinks that contain sugar). If you eat/drink sugar drinks outside the times shown, please add appropriate boxes

| 7am | 8 | 9 | 10 | 11 | 12 | 1pm | 2 | 3 | 4 | 5 | 6 | 7 | 8 | 9 | 10 | 11 | 12am |
| --- | --- | --- | --- | --- | --- | --- | --- | --- | --- | --- | --- | --- | --- | --- | --- | --- | --- |
|  |  |  |  |  |  |  |  |  |  |  |  |  |  |  |  |  |  |

**On your *normal* (non-5:2) weekday:**

Please mark with ticks the hours when you typically eat/drink (eating anything at all, however small, counts, including drinks that contain sugar). If you eat/drink sugar drinks outside the times shown, please add appropriate boxes

| 7am | 8 | 9 | 10 | 11 | 12 | 1pm | 2 | 3 | 4 | 5 | 6 | 7 | 8 | 9 | 10 | 11 | 12am |
| --- | --- | --- | --- | --- | --- | --- | --- | --- | --- | --- | --- | --- | --- | --- | --- | --- | --- |
|  |  |  |  |  |  |  |  |  |  |  |  |  |  |  |  |  |  |

**5:2 *–* 6-months follow-up**

Today’s date_____________ Weight ________ kg Blood pressure _______________ mmHg

Are you currently using any ‘non-study’ weight control methods, products or medications? Yes No

**If yes**, what are you using? _________________________________________________________

How many weeks did you complete the 5-2 for? ____

Are you still on the 5-2 plan? Yes No

**If No,** what was the main reason you stopped? __________________________________

**If Yes,** please complete the questions below:

On average, how many calories do you consume on a fast day? ______ calories

How ready are you to continue with the fast days?: (1=not at all to 10=completely ready) ______

How difficult is it to stick to 5:2 these days? (1=not at all difficult to 10= extremely difficult) ____

How hungry are you on the fast days? (1=not at all hungry to 10=extremely hungry) _____

What type of food do you use on the fast days? Pre-packaged Home cooked Both

Compared to before you started the 5-2 diet, are the fast days now:

**Easier More difficult The same difficulty** to complete (circle one)

On your fast days, is there a part of the day when you feel more hungry or uncomfortable than at

other times of the day?:

| MORNING | AFTERNOON | EVENING | AT NIGHT | NO CLEAR DIFFERENCE |
| --- | --- | --- | --- | --- |

Have there been any changes to your health or medications since you joined the study? Yes No

**If Yes,** please list them here: ________________________________________________________

**On your 5-2 days:**

Please mark with ticks the hours when you typically eat/drink (eating anything at all, however small, counts, including drinks that contain sugar). If you eat/drink sugar drinks outside the times shown, please add appropriate boxes

| 7am | 8 | 9 | 10 | 11 | 12 | 1pm | 2 | 3 | 4 | 5 | 6 | 7 | 8 | 9 | 10 | 11 | 12am |
| --- | --- | --- | --- | --- | --- | --- | --- | --- | --- | --- | --- | --- | --- | --- | --- | --- | --- |
|  |  |  |  |  |  |  |  |  |  |  |  |  |  |  |  |  |  |

**On your *normal* (non-5:2) weekday:**

Please mark with ticks the hours when you typically eat/drink (eating anything at all, however small, counts, including drinks that contain sugar). If you eat/drink sugar drinks outside the times shown, please add appropriate boxes

| 7am | 8 | 9 | 10 | 11 | 12 | 1pm | 2 | 3 | 4 | 5 | 6 | 7 | 8 | 9 | 10 | 11 | 12am |
| --- | --- | --- | --- | --- | --- | --- | --- | --- | --- | --- | --- | --- | --- | --- | --- | --- | --- |
|  |  |  |  |  |  |  |  |  |  |  |  |  |  |  |  |  |  |

**INTERNATIONAL PHYSICAL ACTIVITY QUESTIONNAIRE**

The questions below ask about the **last 7 days**. Please think about the activities you do at work, at home, to get from

place to place, and in your spare time for recreation, exercise or sport.

1. Think about all the **vigorous** activities that you did in the **last 7 days**. This refers to activities that take hard

physical effort and make you breathe much harder than normal. Think *only* about those physical activities that

you did for at least 10 minutes at a time.

a) During the **last 7 days**, on how many days did you do **vigorous** physical activities like heavy lifting, digging, aerobics, or fast cycling?

_____ **days per week**

No vigorous physical activities ***Skip to question 2***

How much time did you usually spend doing **vigorous** physical activities on one of those days?

_____ **hours per day** _____ **minutes per day** Don’t know/Not sure

1. Now, think about all the **moderate** activities that you did in the **last 7 days**. **Moderate** activities refer to activities

that take moderate effort and make you breathe somewhat harder than normal. Think only about those physical

activities that you did for at least 10 minutes at a time.

a) During the **last 7 days,** on how many days did you do **moderate** physical activities like carrying light loads,

cycling at a regular pace or doubles tennis? Do not include walking.

_____ **days per week**

No moderate physical activities ***Skip to question 3***

How much time did you usually spend doing **moderate** physical activities on one of those days?

_____ **hours per day** _____ **minutes per day** Don’t know/Not sure

1. Now, think about the time you spent **walking** in the **last 7 days**. This includes at work and at home, walking to

travel from place to place, and any other walking that you might do solely for recreation, sport, exercise, or leisure.

a) During the **last 7 days**, on how many days did you **walk** for at least 10 minutes at a time?

_____ **days per week**

No walking ***Skip to question 4***

b) How much time did you usually spend **walking** on one of those days?

_____ **hours per day** _____ **minutes per day** Don’t know/Not sure

1. The last question is about the time you spent **sitting** on weekdays during the **last 7 days**. Include time spent

at work, at home, while doing course work and during leisure time. This may include time spent sitting at a desk,

visiting friends, reading, or sitting or lying down to watch television.

During the **last 7 days**, how much time did you spend **sitting** on a **week day**?

_____ **hours per day** _____ **minutes per day** Don’t know/Not sure

**FAT AND FIBER QUESTIONNAIRE**

For each of the questions below, please tick **one** response that most applies to you

| In the past three months how often did you… | | | | |
| --- | --- | --- | --- | --- |
|  | Usually or always | Often | Sometimes | Rarely or never |
| Eat frozen yoghurt or sorbet instead of ice cream? |  |  |  |  |
| Use low-calorie salad dressing instead of regular? |  |  |  |  |
| Eat low-fat cheese instead of regular cheese? |  |  |  |  |
| Drink skimmed or semi-skimmed milk instead of whole? |  |  |  |  |
| Use low-fat spray oil instead of oil, margarine or butter? |  |  |  |  |
| Eat a potato without butter or margarine? |  |  |  |  |
| Eat bread with butter or margarine? |  |  |  |  |
| Put butter or margarine on vegetables? |  |  |  |  |
| Take the skin off chicken? |  |  |  |  |
| Eat baked or boiled chicken? |  |  |  |  |
| Trim visible fat from your meat? |  |  |  |  |
| Eat a small portion of meat? (e.g. the size of a deck of cards) |  |  |  |  |
| Eat baked or broiled fish? |  |  |  |  |
| Eat raw vegetables for a snack? |  |  |  |  |
| Eat two or more vegetables at dinner? |  |  |  |  |
| Eat a vegetable at lunch? |  |  |  |  |
| Eat fruit for dessert? |  |  |  |  |
| Eat a vegetarian dinner? |  |  |  |  |
| Eat meatless pasta sauce? |  |  |  |  |
| Eat fish or chicken instead of red meat? |  |  |  |  |
| Eat high-fiber cereals? e.g bran flakes |  |  |  |  |
| Eat cereal (hot or cold) for breakfast? |  |  |  |  |
| Eat whole-grain crackers or bread? |  |  |  |  |
| Add bran to casseroles or cereal? |  |  |  |  |
| Eat raw vegetables for snacks instead of crisps? |  |  |  |  |
| Eat fruit for breakfast? |  |  |  |  |
| Eat whole-wheat instead of regular pasta? |  |  |  |  |
| Eat brown rice instead of white rice? |  |  |  |  |

**5:2 *–* 12-months follow-up**

Today’s date_____________ Weight ________ kg Blood pressure _______________ mmHg

Are you currently using any ‘non-study’ weight control methods, products or medications? Yes No

**If yes**, what are you using? _________________________________________________________

How long did you complete the 5-2 for? ____ weeks/months

Are you still on the 5-2 plan? Yes No

**If No,** what was the main reason you stopped? __________________________________

**If Yes,** please complete the questions below:

On average, how many calories do you consume on a fast day? ______ calories

How ready are you to continue with the fast days?: (1=not at all to 10=completely ready) ______

How difficult is it to stick to 5:2 these days? (1=not at all difficult to 10= extremely difficult) ____

How hungry are you on the fast days? (1=not at all hungry to 10=extremely hungry) _____

What type of food do you use on the fast days? Pre-packaged Home cooked Both

Compared to before you started the 5-2 diet, are the fast days now:

**Easier More difficult The same difficulty** to complete (circle one)

On your fast days, is there a part of the day when you feel more hungry or uncomfortable than at

other times of the day?:

| MORNING | AFTERNOON | EVENING | AT NIGHT | NO CLEAR DIFFERENCE |
| --- | --- | --- | --- | --- |

Have there been any changes to your health or medications since you joined the study? Yes No

**If Yes,** please list them here: ________________________________________________________

**On your 5-2 days:**

Please mark with ticks the hours when you typically eat/drink (eating anything at all, however small, counts, including drinks that contain sugar). If you eat/drink sugar drinks outside the times shown, please add appropriate boxes

| 7am | 8 | 9 | 10 | 11 | 12 | 1pm | 2 | 3 | 4 | 5 | 6 | 7 | 8 | 9 | 10 | 11 | 12am |
| --- | --- | --- | --- | --- | --- | --- | --- | --- | --- | --- | --- | --- | --- | --- | --- | --- | --- |
|  |  |  |  |  |  |  |  |  |  |  |  |  |  |  |  |  |  |

**On your *normal* (non-5:2) weekday:**

Please mark with ticks the hours when you typically eat/drink (eating anything at all, however small, counts, including drinks that contain sugar). If you eat/drink sugar drinks outside the times shown, please add appropriate boxes

| 7am | 8 | 9 | 10 | 11 | 12 | 1pm | 2 | 3 | 4 | 5 | 6 | 7 | 8 | 9 | 10 | 11 | 12am |
| --- | --- | --- | --- | --- | --- | --- | --- | --- | --- | --- | --- | --- | --- | --- | --- | --- | --- |
|  |  |  |  |  |  |  |  |  |  |  |  |  |  |  |  |  |  |

**INTERNATIONAL PHYSICAL ACTIVITY QUESTIONNAIRE**

The questions below ask about the **last 7 days**. Please think about the activities you do at work, at home, to get from

place to place, and in your spare time for recreation, exercise or sport.

1. Think about all the **vigorous** activities that you did in the **last 7 days**. This refers to activities that take hard

physical effort and make you breathe much harder than normal. Think *only* about those physical activities that

you did for at least 10 minutes at a time.

a) During the **last 7 days**, on how many days did you do **vigorous** physical activities like heavy lifting, digging, aerobics, or fast cycling?

_____ **days per week**

No vigorous physical activities ***Skip to question 2***

How much time did you usually spend doing **vigorous** physical activities on one of those days?

_____ **hours per day** _____ **minutes per day** Don’t know/Not sure

1. Now, think about all the **moderate** activities that you did in the **last 7 days**. **Moderate** activities refer to activities

that take moderate effort and make you breathe somewhat harder than normal. Think only about those physical

activities that you did for at least 10 minutes at a time.

a) During the **last 7 days,** on how many days did you do **moderate** physical activities like carrying light loads,

cycling at a regular pace or doubles tennis? Do not include walking.

_____ **days per week**

No moderate physical activities ***Skip to question 3***

How much time did you usually spend doing **moderate** physical activities on one of those days?

_____ **hours per day** _____ **minutes per day** Don’t know/Not sure

1. Now, think about the time you spent **walking** in the **last 7 days**. This includes at work and at home, walking to

travel from place to place, and any other walking that you might do solely for recreation, sport, exercise, or leisure.

a) During the **last 7 days**, on how many days did you **walk** for at least 10 minutes at a time?

_____ **days per week**

No walking ***Skip to question 4***

b) How much time did you usually spend **walking** on one of those days?

_____ **hours per day** _____ **minutes per day** Don’t know/Not sure

1. The last question is about the time you spent **sitting** on weekdays during the **last 7 days**. Include time spent

at work, at home, while doing course work and during leisure time. This may include time spent sitting at a desk,

visiting friends, reading, or sitting or lying down to watch television.

During the **last 7 days**, how much time did you spend **sitting** on a **week day**?

_____ **hours per day** _____ **minutes per day** Don’t know/Not sure

**FAT AND FIBER QUESTIONNAIRE**

For each of the questions below, please tick **one** response that most applies to you

| In the past three months how often did you… | | | | |
| --- | --- | --- | --- | --- |
|  | Usually or always | Often | Sometimes | Rarely or never |
| Eat frozen yoghurt or sorbet instead of ice cream? |  |  |  |  |
| Use low-calorie salad dressing instead of regular? |  |  |  |  |
| Eat low-fat cheese instead of regular cheese? |  |  |  |  |
| Drink skimmed or semi-skimmed milk instead of whole? |  |  |  |  |
| Use low-fat spray oil instead of oil, margarine or butter? |  |  |  |  |
| Eat a potato without butter or margarine? |  |  |  |  |
| Eat bread with butter or margarine? |  |  |  |  |
| Put butter or margarine on vegetables? |  |  |  |  |
| Take the skin off chicken? |  |  |  |  |
| Eat baked or boiled chicken? |  |  |  |  |
| Trim visible fat from your meat? |  |  |  |  |
| Eat a small portion of meat? (e.g. the size of a deck of cards) |  |  |  |  |
| Eat baked or broiled fish? |  |  |  |  |
| Eat raw vegetables for a snack? |  |  |  |  |
| Eat two or more vegetables at dinner? |  |  |  |  |
| Eat a vegetable at lunch? |  |  |  |  |
| Eat fruit for dessert? |  |  |  |  |
| Eat a vegetarian dinner? |  |  |  |  |
| Eat meatless pasta sauce? |  |  |  |  |
| Eat fish or chicken instead of red meat? |  |  |  |  |
| Eat high-fiber cereals? e.g bran flakes |  |  |  |  |
| Eat cereal (hot or cold) for breakfast? |  |  |  |  |
| Eat whole-grain crackers or bread? |  |  |  |  |
| Add bran to casseroles or cereal? |  |  |  |  |
| Eat raw vegetables for snacks instead of crisps? |  |  |  |  |
| Eat fruit for breakfast? |  |  |  |  |
| Eat whole-wheat instead of regular pasta? |  |  |  |  |
| Eat brown rice instead of white rice? |  |  |  |  |
