# Supplementary material for: A randomised controlled trial of the 5:2 diet
Source: PLoS One. 2021 Nov 17;16(11):e0258853. doi: 10.1371/journal.pone.0258853 (PMC8598045; doi:10.1371/journal.pone.0258853)
Supplement: S6 File — (DOCX) [file pone.0258853.s010.docx]

# Counting Calories

Many supermarket foods have the number of calories contained shown on **food labels** like this:


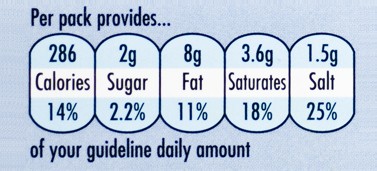


You need to check if the number shows calories in the whole package (as above) or in just a portion (eg 100 grams). For example, in the label below, the number of calories refers to the amount found in *half* a pizza. If you were to eat the whole pizza, you would need to double the number of calories in your count.


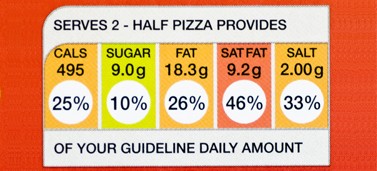


You **do not need to count vegetables or salad** as part of your calorie allowance. You should count any dressings you use though, and any oils that you cook with.

There are books and websites which provide caloric contents of practically any food item, for example:

- <http://www.weightlossresources.co.uk/>
- <https://www.myfitnesspal.com/>
- <http://www.nhs.uk/Livewell/weight-loss-guide/Pages/calorie-counting.aspx>
