# Supplementary material for: A randomised controlled trial of the 5:2 diet
Source: PLoS One. 2021 Nov 17;16(11):e0258853. doi: 10.1371/journal.pone.0258853 (PMC8598045; doi:10.1371/journal.pone.0258853)
Supplement: S7 File — (DOC) [file pone.0258853.s011.doc]

**Some suggestions for introducing exercise into your life**

Some people enjoy going to the gym or attending an exercise class but others find it difficult to fit these options into their lifestyle for a variety of reasons. Here are some other options.

**1.** If you can do walks, you can try **faster or longer walking** as your more vigorous workout.

There are a number of walking groups across London. For more information about walking as a form of exercise or to search for your local walking group visit: https://www.walkingforhealth.org.uk. The vast majority of walking groups are free to attend, with some occasionally asking for a small donation.

**2.** An exercise DVD or video can be a good option. The **‘10-minute solution’** range of DVDs is good. They cost around £5 on amazon.co.uk. The series include Pilates, Yoga, Fitness Ball, Fat blasting, Dancing and other types of exercise so you can find one that you like.

**3.** If you have a **Wii or Wii-fit** at home, this is great and can be done with other members of the family.

**4. Water-aerobics** is suitable even if you have physical restrictions or disabilities. You do not need to be able to swim to take part. It’s a great way of enabling you to exercise without putting excess pressure on your joints.

**Get Active London** provide a London-wide database of aqua aerobics clubs. You can search for your local club here: http://getactivelondon.com.

**5.** If you are able to join an exercise class, **Get Active London** provides a database of London-wide exercise classes. The list ranges from Zumba to body conditioning, and everything in-between!

You can search for what’s available in your local area here: http://www.getactivelondon.com or find out more by emailing info@getactivelondon.com.

**6.** Another great way of getting more active is through joining a gym. GLL, a not-for-profit charitable social enterprise, have ‘Better’ leisure centers based all around the UK.

They are very affordable, inclusive, and provide a range of activities (so there’s something for everyone!).

To find out more about Better gyms you can visit http://www.better.org.uk.
