# Supplementary material for: A randomised controlled trial of the 5:2 diet
Source: PLoS One. 2021 Nov 17;16(11):e0258853. doi: 10.1371/journal.pone.0258853 (PMC8598045; doi:10.1371/journal.pone.0258853)
Supplement: S8 File — (DOCX) [file pone.0258853.s012.docx]

**A GUIDE TO THE 5:2 DIET**

**What is 5:2?** When we want to lose weight, we normally ‘go on a diet’ and try to eat less. The problem is that for most of us, eating less than we want to is accompanied by discomfort and hunger and this makes it difficult to maintain the regime. After a while, the weight we managed to lose creeps back.

The 5:2 diet represents a different and promising approach. All the dieting is done on only two days of the week (separated by two or three days). On the ‘low calorie days’ (we shall call them ‘fast days’ for simplicity though they do not involve total fasting), women consume only 500 calories while men are allowed 600. For the remaining five days, you eat normally.

There are several reasons why the 5:2 diet is likely to be good for our health and for weight loss. From the point of view of a dieter, its most important features are its simplicity, and the fact that it only requires effort for one day at a time. Once that is over and done with, there are two or three days ahead when little effort is required.

**How do you start?** Starting the 5:2 diet is simple. Decide on your first fast day and prepare or buy the food you are going to eat on that day.

**Practical tips:** Here are six tips from people who used the 5:2 diet successfully:

1.Most dieters find the first few fast days difficult, and some give up. Successful users of the 5:2 learn to tolerate the fast days by sticking to the diet, hungry or not, for the first few weeks, after which things get much easier. Knowing this may help you to persevere over this initial difficult period.

2. Prepare the food you are going to eat on the fast day in advance. Below is guidance on 5:2 meals. **Perhaps the simplest approach is to buy 5:2 meals ready-made in Superdrug.** Some dieters use the same food every time to keep things simple.

3. Allocate the low-calorie days to days when you are busy. Distraction is the best way of coping with hunger.

4. Many find it useful to stick to the same days of the week so they develop a standard routine, only switching to a different day if needed (e.g. if there is a pre-scheduled party or dinner).

5. Most people eat their food allowance as breakfast and supper, but some space food regularly during the day. Experiment to find a schedule most comfortable for you.

6. There are several internet sites where 5:2 dieters share tips, recipes, advice and experience. These can be helpful for finding information and maintaining interest and motivation. Some are listed below.

**Some resources you may find helpful**

***Books:***

Mosley M & Spencer M. The fast diet, 2013 (£3 on Amazon.co.uk)

Harrison K. The 5:2 diet book, 2012 (£5 on Amazon.co.uk)

***Blogs and users groups:***

<http://thefastdiet.co.uk/forums/>

<https://www.facebook.com/groups/the52diet/>

***Recipe ideas:***

<http://www.52recipes.co.uk/>

***Apps:***

MyFitnesspal (free)

5:2 Complete Meal Planner (£1.99)

**5:2 meal examples**

To give you an idea of meals adding up to about 500 or 600 kcal, here are five examples of home cooked and pre-packaged foods divided into breakfast, lunch, supper and snacks. Some dieters eat regularly, some prefer to have more substantial breakfasts, some save everything for the evening – do experiment to find out what works best for you.

| **Breakfast** | **Breakfast** |
| --- | --- |
| Spinach omelette (110 kcal)  1 medium egg, 60g fresh spinach, spray oil (eg. ‘fry light’) | Low-fat greek yoghurt and apricot (70 kcal)    25g yoghurt and 2 chopped apricots |
| **Lunch** | **Lunch** |
| Grilled chicken breast salad (180 kcal)  1 chicken breast, without skin, grilled with spray oil, 40g mixed leaves, 5 cherry tomatoes halved, lemon juice | Tomato and Lentil Soup (120 kcal)  150g lentils, vegetable stock, garlic, ginger, spices and 1 can chopped tomatoes |
| **Dinner** | **Dinner** |
| Prawn Stir fry (210 kcal)  150g prawns stir-fried with mixed veg, ½ sliced red chilli, 1 tsp oil, 1 clove of garlic & 1 tsp soy sauce. | Vegetable Spaghetti (240 kcal)  50g spaghetti, 1 pepper, 50g spinach, ½ tsp garlic puree |
| **Snacks** | **Snacks** |
| 2 satsumas (40 kcal) | Mini milk ice lolly (30 kcal) |

| **Breakfast (50p)**  Activia 0% fat Yoghurt (70 kcal) | **Breakfast (20p)**  Porridge sachet with water 27g (100 kcal) | **Breakfast (20p)**  Banana (100 kcal) |
| --- | --- | --- |
| **Lunch (85p)**  Weight Watchers Chicken Noodle Soup (51 kcal) and one rice cake (30 kcal) | **Lunch (£1.35)**  Tesco Sushi Taster (110 kcal) | **Lunch (85p)**  Two rice cakes (60 kcal) and one cheese triangle (25 kcal) |
| **Dinner (£3.50)**  M&S Count on Us - Chicken Mini Fillet In A Red Wine & Mushroom Sauce With Cabbage & Spring Onion Mash (300 kcal) | **Dinner (£3.90)**  Innocent Mexican Chipotle Chilli Vegetable Pot (285 kcal) | **Dinner (£3.00)**  Sainsbury’s Chicken Tikka Biryani (316 kcal) |
| **Snacks (25p)**  Sainsbury’s popcorn (52 kcal)    **TOTAL: £5.10** | **Snacks (70p)**  Hartley’s jelly (10 kcal)    **TOTAL: £6.15** | **Snacks (30p)**  Two satsumas (40 kcal)  **TOTAL: £4.35** |

Drinks with milk will use up calories that could be used elsewhere. Water, diet drinks, black coffee or herbal teas are fine.

Vegetables, fish, eggs, lean meat and soups are low in calories so you can eat more compared to high calorie foods.

**What to do on the five non-fasting days?**

**Eat:** You do not need to limit your food intake on the non-fasting days. Although you are likely to eat slightly more on the day following the fast day, people normally do not overeat much (the fast days can also reduce appetite).

**Walk/exercise:** Aim to walk 10,000 steps per day. Don’t worry if you’re not achieving 10,000 steps immediately, set small targets so you gradually increase your walking every week. Wearing a pedometer is the best way to monitor your number of steps. We recommend the *Nakosite Best Walking 3D Pedometer (£12.99),* but there are cheaper ones e.g. on Amazon.com. You can also download free apps on your phone, such as *Pacer*.

**Weigh yourself:** It is also a good idea to have a set of scales at home and to monitor your weight regularly. Weigh yourself at the same time of day on a hard, flat surface.

**Simple changes:** Making small changes to your diet and habits can also help. This could be a switch from whole milk to semi-skimmed, from normal to diet coke, getting used to tea and coffee without sugar, removing biscuits and peanuts from your living room, etc.

**Do not eat when you are not hungry:** Before you are about to eat, check if you are hungry. If not, do not eat. Similarly, stop eating when you are full.

**Say NO to unnecessary food**: We often eat snacks just because they are there or because someone is offering them to us. Pat yourself on the shoulder every time you manage to say No to unnecessary food.

**Good luck with your programme!**
